# Architectural good practices for reproducible benchmarking in protein machine learning

**DOI:** 10.3389/fbinf.2026.1925740

**Published:** 2026-09-08

**Authors:** Julián García-Vinuesa, Diego Fernández-Villegas, Michelle Soto-García, Xavier Cadet, Mehdi D. Davari, Frederic Cadet, Juan A. Asenjo, Roberto Uribe-Paredes, David Medina-Ortiz

**Affiliations:** 1 Departamento de Ingeniería En Computación, Universidad de Magallanes, Punta Arenas, Chile; 2 Dartmouth College, Hanover, NH, United States; 3 Department of Bioorganic Chemistry, Leibniz Institute of Plant Biochemistry, Halle, Germany; 4 AI for Biologics, PEACCEL, Paris, France; 5 Departamento de Ingeniería Química, Biotecnología y Materiales, Universidad de Chile, Santiago, Chile

**Keywords:** computational infrastructure, FAIR workflows, good practices, interoperability, protein machine learning, reproducible benchmarking, verifiable workflows, workflow provenance

## Abstract

Reliable benchmarking in protein machine learning requires biological tasks, datasets, representations, execution conditions, and evaluation regimes to be explicitly defined and traceable. Protein-specific dependencies, including label semantics, negative-class construction, evolutionary relationships, similarity control, inferred structures, protein language model configurations, and potential pretraining contamination, can alter benchmark interpretation. This Perspective provides an operational, protein-specific synthesis of established reproducibility practices through an artefact-linked architecture. We define five core requirements for verifiable benchmarking, including deterministic curation, persistent and versioned artefacts, explicit interface contracts, reproducible execution, and transparent evaluation. Prediction provenance, uncertainty, calibration, and explainability are treated as an additional decision-readiness layer. These requirements are translated into minimum records, provenance links, and verification checks. An antimicrobial peptide classification case study demonstrates their application to alternative task formulations, negative-class definitions, dataset compositions, and partitioning strategies within a controlled workflow.

## 1 Introduction

Machine learning is now central to protein science, supporting structure prediction, variant effect modelling, functional annotation, and *de novo* protein design (Kim et al., 2025; Sun and Shen, 2025; Jendrusch et al., 2025). Advances in biological data resources, representation learning, geometric modelling, and protein foundation models have expanded the range of computationally accessible tasks (Weissenow and Rost, 2025; García-Vinuesa et al., 2025; Lin et al., 2025; Consortium, 2025). [However, their reliability depends not only on predictive architectures, but also on how biological data and computational artefacts are curated, transformed, partitioned, executed, and evaluated (Kapoor and Narayanan, 2023). Studies addressing similar tasks may therefore differ in dataset construction, redundancy control, representation extraction, model selection, and evaluation protocols] (Martinez et al., 2025; Kucera et al., 2023; Kouba et al., 2023). When these [decisions are undocumented or distributed across scripts, configuration files, and software environments, results become difficult to reproduce, compare, audit, or trace to their provenance (Antunes and Hill, 2024; Gundersen et al., 2022; Leipzig et al., 2021; Pimentel et al., 2019; Kapoor and Narayanan, 2023).

These dependencies are particularly consequential in protein machine learning because protein sequences are evolutionarily related and many workflow inputs are computationally derived. Predicted structures, similarity control, functional annotations, and representation settings (Abramson et al., 2024; Ferrer Florensa et al., 2024; Char et al., 2025; Lin et al., 2023) can alter the effective prediction problem, the independence of evaluation data, and the biological interpretation of reported performance (Kapoor and Narayanan, 2023; Herrera-Rocha et al., 2025; Bernett et al., 2024).

The consequences are especially visible in benchmarking, where meaningful comparison requires more than common datasets and metrics (Zhang et al., 2026; Semmelrock et al., 2025). Resources such as EnzEngDB (L et al., 2026), CombinGym (Chen et al., 2026), ProteinGym (Notin et al., 2023), and FLIP (Dallago et al., 2021) provide curated tasks and evaluation settings, while EnzymeML (Lauterbach et al., 2023), Codabench (Xu et al., 2022), OpenML (Feurer et al., 2021), Snakemake (Köster and Rahmann, 2012), Nextflow (Di Tommaso et al., 2017), and related initiatives (Fursin et al., 2020; Swainston et al., 2018; Amstutz et al., 2016) support structured reporting, portable execution, and reproducible evaluation. Nevertheless, these resources generally address complementary parts of the workflow, while dependencies between task definition, curation, partitioning, representation generation, execution, and evaluation frequently remain fragmented (Herrera-Rocha et al., 2025; Cheng et al., 2026; Graber et al., 2025; Heil et al., 2021). The remaining gap is an integrated way to make these protein-specific dependencies explicit and verifiable across the workflow.

This Perspective provides an operational, protein-specific synthesis of established reproducibility practices (Wilkinson et al., 2016) through an artefact-linked architecture connecting data provenance (Schlegel and Sattler, 2025), similarity-aware generalisation (Ferrer Florensa et al., 2024), representation extraction, controlled execution, and benchmark interpretation (Herrera-Rocha et al., 2025; Semmelrock et al., 2025). We organise the recommendations around five core requirements, including deterministic curation, persistent and versioned artefacts, explicit interface contracts, reproducible execution, and transparent evaluation. Uncertainty, calibration, explainability, and prediction provenance are treated as an additional decision-readiness layer for workflows guiding biological or experimental actions. A demonstrative antimicrobial peptide classification case study illustrates how task definition, negative-class construction, dataset composition, and partitioning can reshape apparent performance under controlled representation, model, and evaluation settings. By treating dataset semantics and workflow assumptions as part of the scientific result, this Perspective aims to support more reproducible, comparable, and biologically interpretable protein machine learning benchmarks.

## 2 The infrastructure bottleneck in protein machine learning

The infrastructure bottleneck lies in preserving the biological meaning, configuration, and provenance of workflow objects as they move between stages. When these relationships are implicit, apparently comparable analyses may no longer represent the same task, inputs, or evaluation regime (Herrera-Rocha et al., 2025; Draizen et al., 2024; Kapoor and Narayanan, 2023; Bernett et al., 2024).

A protein dataset is not fully specified by sequences and labels alone. Database releases, retrieval queries, inclusion criteria, annotation rules, duplicate handling, label harmonisation, and ambiguous or missing annotations determine the effective prediction task (Consortium, 2025; Pop et al., 2025; Helmy et al., 2016; Youngs et al., 2014). Negative-class construction is particularly consequential because experimentally supported negatives, unannotated sequences, background proteins, and artificial decoys encode different biological questions and may introduce distinct compositional or physicochemical biases.

Evolutionary relationships add another constraint because protein sequences are not independent observations. Homologous or near-duplicate sequences shared across development and evaluation sets can inflate performance (Ferrer Florensa et al., 2024; Chen et al., 2021; Bushuiev et al., 2024), while similarity thresholds, clustering rules, and partitioning strategies determine the generalisation regime being tested (Chiu and Ong, 2022; Bonin et al., 2023; Petti and Eddy, 2022; Joeres et al., 2025). Random, redundancy-controlled, family-level, temporal, and structure-aware partitions probe distinct regimes and should therefore not be treated as interchangeable benchmark settings (Petti and Eddy, 2022; Ferrer Florensa et al., 2024; Zhou et al., 2019; Aguilera-Puga and Plisson, 2024).

Workflow inputs may also be computationally derived. Homology searches and alignments depend on database releases, software, thresholds, and parameters (Consortium, 2025; Chiu and Ong, 2022; Wei et al., 2023); structure-based workflows inherit the provenance and limitations of experimental or predicted structures (Abramson et al., 2024; Terwilliger et al., 2024); and protein language-model representations depend on checkpoint, layer selection, tokenisation, pooling, and preprocessing (Li et al., 2022; Dota et al., 2024; Hoang and Singh, 2025; Li et al., 2024). Pretraining contamination should also be considered because evaluation sequences or close homologues may have been encountered during foundation-model pretraining even when supervised partitions do not overlap (Hermann et al., 2024).

Even with a fixed biological task, workflow behaviour can change through dependency resolution, numerical precision, hardware, random seeds, nondeterministic operations, or incompatible artefact versions (Gundersen et al., 2022; Leipzig et al., 2021). Outputs from different tools are also not automatically interoperable: an artefact must preserve its meaning, configuration, version, and provenance, not merely use a readable format (Khan et al., 2019). Workflows assembled from scripts, notebooks, local files, and tool-specific configurations may therefore function within one laboratory while lacking the records required for independent inspection and reuse (Pimentel et al., 2019; Heil et al., 2021; Khan et al., 2019; Xiang et al., 2025; Fernández-Díaz et al., 2024).

Here, repeatability refers to rerunning an analysis under equivalent conditions, whereas reproducibility refers to obtaining consistent results through an independent execution using the reported materials and procedures. Auditability requires reconstructing the inputs, transformations, configurations, and executions that produced a result, while verifiability additionally requires explicit checks of artefact integrity, compatibility, provenance, partition constraints, and test isolation. Comparability depends on aligned task definitions, generalisation regimes, and evaluation protocols; interoperability requires interfaces that preserve artefact meaning and provenance; and benchmarkability requires evaluation under a documented and biologically interpretable generalisation regime.

These dependencies cannot be resolved through algorithmic innovation alone. They require explicit records, provenance links, compatibility rules, and validation procedures that remain stable across tools and environments (Graber et al., 2025; Heil et al., 2021; Kapoor and Narayanan, 2023; Bernett et al., 2024; Wilkinson et al., 2016). The following section translates these needs into operational requirements for verifiable protein machine-learning workflows.

## 3 Principles for verifiable protein machine learning

**FIGURE 1:**
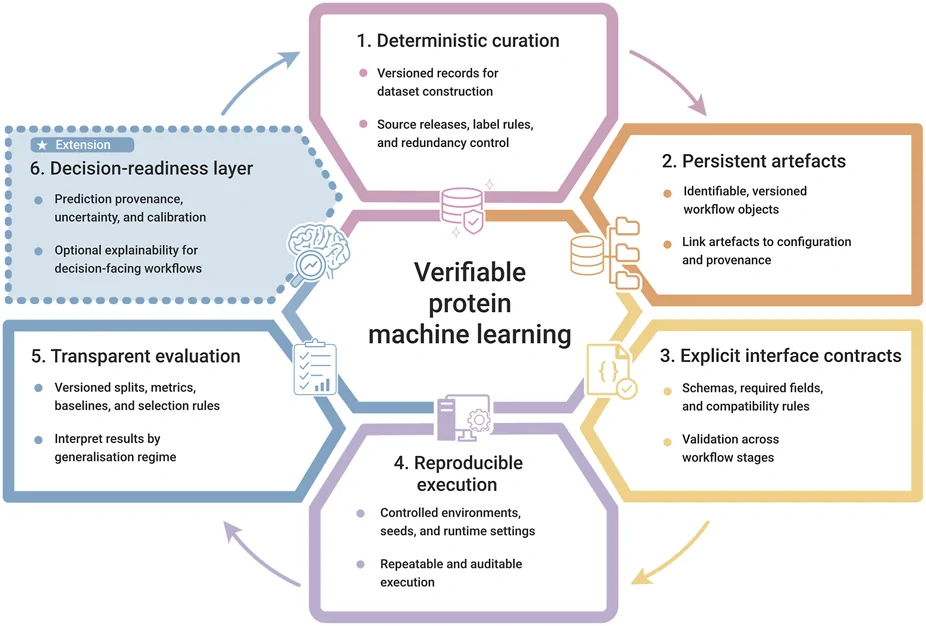
Architectural requirements for verifiable protein machine learning. Five core requirements form the reproducibility architecture: deterministic curation, persistent artefacts, explicit interface contracts, reproducible execution, and transparent evaluation. A decision-readiness layer extends this architecture when workflow outputs guide biological or experimental actions, incorporating prediction provenance, uncertainty, calibration, and, where appropriate, explainability. Connections between components indicate that configuration, compatibility, and provenance must remain traceable across workflow stages.

Building on established guidance for methodological reporting, FAIR data practices, controlled execution, and transparent benchmark design, we translate these practices into an operational architecture for protein machine learning] (Herrera-Rocha et al., 2025; Bernett et al., 2024; Heil et al., 2021; Wilkinson et al., 2016; Longjohn et al., 2024; Petti and Eddy, 2022). Within this architecture, verifiability requires each workflow object to retain sufficient records, provenance relationships, and checks to establish its integrity, compatibility, and declared methodological properties. We distinguish five core requirements for reproducible benchmarking and an additional decision-readiness layer for workflows whose outputs guide biological or experimental actions (Figure 1). Their operational implementation is summarised in Table 1, while Section S1 of the Supplementary Material provides detailed requirements and a minimum compliance checklist.

**TABLE 1:** Operational requirements for verifiable protein machine learning workflows.

Requirement	Protein-specific risk	Minimum required record	Verification check	Implementation and extension
Deterministic curation	Database or annotation drift; ambiguous labels; inappropriate negative classes; redundancy leakage	Source and release; access date; retrieval and filtering rules; sequence and label definitions; inclusion and exclusion criteria; duplicate and redundancy procedures; negative-class construction	Reconstruct the dataset; validate identifiers and sequence alphabets; detect duplicates; confirm class composition and provenance	Machine-readable manifests, dataset cards, FAIR metadata, and versioned curation scripts. Extensions include ontology-supported labels, source comparison, and continuous curation validation
Persistent artefacts	Stale or incompatible datasets, representations, partitions, models, predictions, or results	Persistent identifier; artefact type and version; checksum; generating configuration; execution identifier; upstream and downstream provenance links	Validate checksum integrity, version and schema compatibility, and completeness of artefact lineage	Versioned repositories, persistent identifiers, provenance records, and model or dataset cards. Extensions include immutable archival, retention policies, and automated invalidation of dependent artefacts
Explicit interface contracts	Incompatible identifiers, schemas, dimensions, tokenisation settings, or input–output assumptions	Required fields; data types; dimensions; identifier conventions; allowed values; compatibility constraints; expected input and output behaviour	Validate schemas, dimensions, identifiers, allowed values, and contracts at workflow boundaries	JSON schema, typed interfaces, workflow specifications, and the common workflow language. Extensions include automated contract testing, schema migration rules, and cross-implementation compatibility checks
Reproducible execution	Dependency, hardware, numerical-precision, seed, or runtime drift	Software and dependency versions; environment or container; hardware context; numerical precision; random seeds; runtime parameters; relevant nondeterministic operations	Execute from a clean environment; validate configuration; compare output checksums; test deterministic behaviour where feasible	Workflow engines, containers, lockfiles, environment manifests, and versioned configuration files. Extensions include continuous integration, multi-platform reproduction, and automated output comparison
Transparent evaluation	Leakage; incompatible generalisation regimes; prevalence-dependent metric interpretation; test-set contamination	Exact split assignments; similarity thresholds or grouping rules; split sizes and prevalence; metrics and baselines; model-selection protocol; test-isolation procedure	Check duplicates and homology across partitions; validate prevalence and test isolation; independently recompute reported metrics	Versioned split files, benchmark cards, evaluation manifests, and reusable metric implementations. Extensions include external, temporal, family-level, structure-aware, and prospective evaluation
Decision readiness (extension)	Overconfident predictions or unsupported biological and experimental prioritisation	Sample-level prediction; model, representation, partition, configuration, and execution identifiers; score or probability; uncertainty and calibration records when relevant	Confirm prediction traceability and output completeness; assess calibration and applicability using task-appropriate procedures	Prediction records, model cards, calibration reports, and provenance-aware result tables. Extensions include explanation artefacts, decision logs, and linkage with prospective experimental outcomes

Verifiability begins with deterministic curation, which establishes the biological and computational meaning of the dataset before model development. Dataset construction should be captured through versioned, machine-readable records that preserve sources, transformations, annotation rules, and similarity-control procedures (AlQuraishi, 2019; González-Cebrián et al., 2024; Ferrer Florensa et al., 2024; Youngs et al., 2014).

Datasets and downstream outputs should also remain identifiable beyond the execution in which they were produced. Representations, partitions, trained models, predictions, and evaluation results should therefore be treated as persistent artefacts linked to their versions, generating configurations, execution records, and upstream dependencies (Hartley and Olsson, 2020; Bayarri et al., 2024; Gundersen et al., 2022; Khan et al., 2019). Persistence must be complemented by explicit interface contracts defining identifiers, required fields, data types, dimensions, compatibility conditions, and validation rules before artefacts are consumed downstream (Kanwal et al., 2017; Amstutz et al., 2016; Meyer, 2002).

Stable artefacts and interfaces must be supported by reproducible execution. Software and dependency versions, computational environments, hardware context, numerical precision, random seeds, runtime parameters, and relevant sources of nondeterminism should be recorded with each run (Leipzig et al., 2021). These records should enable clean-environment reruns and associate each result with its generating conditions. Workflow engines such as Nextflow (Di Tommaso et al., 2017), Snakemake (Köster and Rahmann, 2012), and the Common Workflow Language (Amstutz et al., 2016), together with containers, lockfiles, dependency managers, and deterministic seeding, provide practical mechanisms for limiting environmental drift (Kurtzer et al., 2017; Merkel , 2014).

Transparent evaluation closes the core architecture by linking reported performance to a precisely defined benchmark setting. Partition assignments, similarity or grouping rules, class prevalence, metrics, baselines, model-selection procedures, and test-isolation conditions should remain versioned with the evaluation record. This allows results to be interpreted according to the biological generalisation regime actually assessed (Kucera et al., 2023; Ferrer Florensa et al., 2024; Bernett et al., 2024; Pineau et al., 2021; Leipzig et al., 2021).

For decision-facing applications, the architecture can be extended beyond benchmark reproducibility. When predictions are used to prioritise variants, select candidates, or guide experiments (Hie et al., 2020), sample-level outputs should preserve their model, representation, configuration, partition, and execution provenance. Calibration, uncertainty, probabilities, and explanation artefacts should be retained when relevant to the intended decision and supported by the analytical design (Leipzig et al., 2021; Doshi-Velez and Kim, 2017; Hie et al., 2020; Biswas et al., 2021). This extension is not required for every benchmark, but becomes important when computational outputs directly influence biological or experimental actions.

The first five requirements define a minimum basis for reproducible benchmarking, while decision-readiness and other advanced practices can be introduced according to application scale, risk, and reuse expectations. Large or regenerable objects need not always be preserved when they can be reconstructed from stable identifiers, configurations, provenance, and integrity records.

## 4 Failure modes and good-practice recommendations

The operational requirements described above help identify failures that arise when biological meaning, configuration, provenance, or compatibility is lost across task definition, dataset construction, representation generation, model development, execution, and evaluation.

Task-definition and label-semantic drift occur when nominally similar datasets encode different prediction problems. Changes in source data, annotation rules, ambiguous evidence, or negative-class construction (Joshi et al., 2023; Sidorczuk et al., 2022; Pop et al., 2025) can alter class meaning, prevalence, composition, and physicochemical biases, making comparisons misleading when a shared task label conceals distinct biological questions.

A recurrent source of overoptimistic performance in protein machine learning is unintended similarity or dependence between development and evaluation data. Following Kaufman et al. (Kaufman et al., 2012) and its discussion in biological machine learning by Bernett et al. (Bernett et al., 2024), leakage occurs when information unavailable during model development influences training, validation, or performance estimation. In protein machine learning, homologous, near-duplicate, or otherwise non-independent sequences may cross training and test sets when redundancy control, clustering thresholds, metadata filters, or partitioning rules are insufficiently specified (Bushuiev et al., 2024; Bonin et al., 2023). This can inflate performance and overstate biological generalisation. Preventing such leakage, however, does not by itself establish distant biological generalisation. Random, redundancy-controlled, family-level, temporal, and structure-aware partitions preserve different relationships between development and evaluation data and therefore support different performance claims (Ferrer Florensa et al., 2024; Petti and Eddy, 2022; Zhou et al., 2019; Aguilera-Puga and Plisson, 2024).

Even when the underlying protein sequence is unchanged, its computational representation may vary with how it is generated. Protein language-model embeddings can differ with the checkpoint, tokenisation, padding or truncation, layer selection, pooling strategy, implementation, batching, and numerical precision (Li et al., 2024; Vieir et al., 2025), whereas alignments, derived features, and predicted structures depend on database releases, tool or model versions, parameters, and preprocessing procedures (Consortium, 2025; Abramson et al., 2024; Chen et al., 2022; Zheng et al., 2024). If these conditions are not preserved, identical sequences may produce non-equivalent downstream inputs. A related provenance risk is pretraining contamination, because evaluation sequences or close homologues may have been included in a foundation model’s pretraining corpus even when supervised partitions do not overlap (Hermann et al., 2024). Studies should therefore report checkpoint identifiers, available pretraining-corpus provenance, relevant dataset dates, and known sequence-overlap or homology analyses whenever possible.

Execution drift occurs when changes in software dependencies, hardware, random seeds, runtime parameters, numerical precision, or nondeterministic operations alter results across runs, whereas lineage drift arises when outputs can no longer be traced unambiguously to their generating artefacts and configurations (Martinez et al., 2025; Antunes and Hill, 2024; Schlegel and Sattler, 2025). If datasets, representations, partitions, models, or result tables are updated independently, downstream analyses may combine incompatible objects and obscure whether variation originates from the data, modelling choices, or execution conditions (Leipzig et al., 2021; Schlegel and Sattler, 2025). Good practice therefore requires recording the computational state of each run and the provenance relationships connecting its inputs, intermediate artefacts, models, and outputs.

An additional failure arises when benchmark results and workflow artefacts are not maintained as linked and versioned objects. Apparent improvements may reflect changes in preprocessing, partitions, leakage controls, class prevalence, metric implementations, baseline definitions, model-selection procedures, run aggregation, or test-set access rather than substantive methodological progress (Orioli et al., 2020; Xu et al., 2022; Bushuiev et al., 2024). Comparisons should therefore remain traceable to the exact artefact versions, partitions, metric definitions, validation procedures, and test-isolation conditions used. Uncertainty summaries should describe the number and structure of repeated runs, and conclusions should remain limited to the datasets, representations, models, and generalisation regimes actually evaluated.

Decision-readiness failures form a separate category. Reproducible benchmarks do not universally require explanation artefacts, but predictions used to prioritise candidates or guide experiments require stronger traceability. Prediction provenance, calibration, uncertainty, and applicability should be recorded when relevant, while explanations should be included only when supported by the model, analytical design, and intended decision (Karim et al., 2023; Hie et al., 2020; Medina-Ortiz et al., 2025).

These failures are interdependent. Task semantics determine class composition, partitioning defines the generalisation regime, representation and execution drift affect downstream models, and inconsistent evaluation can obscure the origin of performance differences. The following case study examines these dependencies in antimicrobial peptide classification through alternative task formulations, dataset compositions, and partitioning strategies under a controlled representation and modelling setup.

## 5 Demonstrative case study: workflow decisions reshape apparent model performance

To illustrate why benchmark-defining decisions should be treated as part of the scientific result, we examined how alternative task and evaluation choices changed apparent performance in antimicrobial peptide classification. The objective was not to develop a new AMP predictor or rank classifiers, but to show how commonly implicit workflow decisions can alter reported performance and its interpretation.

Two binary classification tasks were constructed from a sequence-level activity matrix derived from Peptipedia v2.0 (Cabas-Mora et al., 2024), containing 86,477 unique canonical peptide sequences of 2–150 residues. Both used the same 34,410 antimicrobial-positive peptides but differed in the negative class, with 10,211 toxic peptides without antimicrobial evidence in the first task and all 52,067 peptides without antimicrobial evidence in the second. These formulations differed in size, prevalence, composition, and biological meaning and therefore represent alternative benchmark constructions rather than simple relabelling of the same observations.

Each task was evaluated under a stratified-random reference and four sequence-similarity-aware regimes, H90, H70, H50, and H30, with complete MMseqs2 (Steinegger and Söding, 2017) groups retained within partitions. A single frozen 320-dimensional ESM2 (Lin et al., 2023) representation was used throughout. Four conventional classifier families were included as analytical strata; one task-specific variant per family was selected under RANDOM validation and then frozen across all partitioning regimes. The analysis comprised 4,000 final model executions from two tasks, five regimes, four model families, and 100 partition seeds. Complete methodological details are provided in SI Section S2, with model-selection outcomes and execution-integrity checks in SI Section S3.3.

**FIGURE 2:**
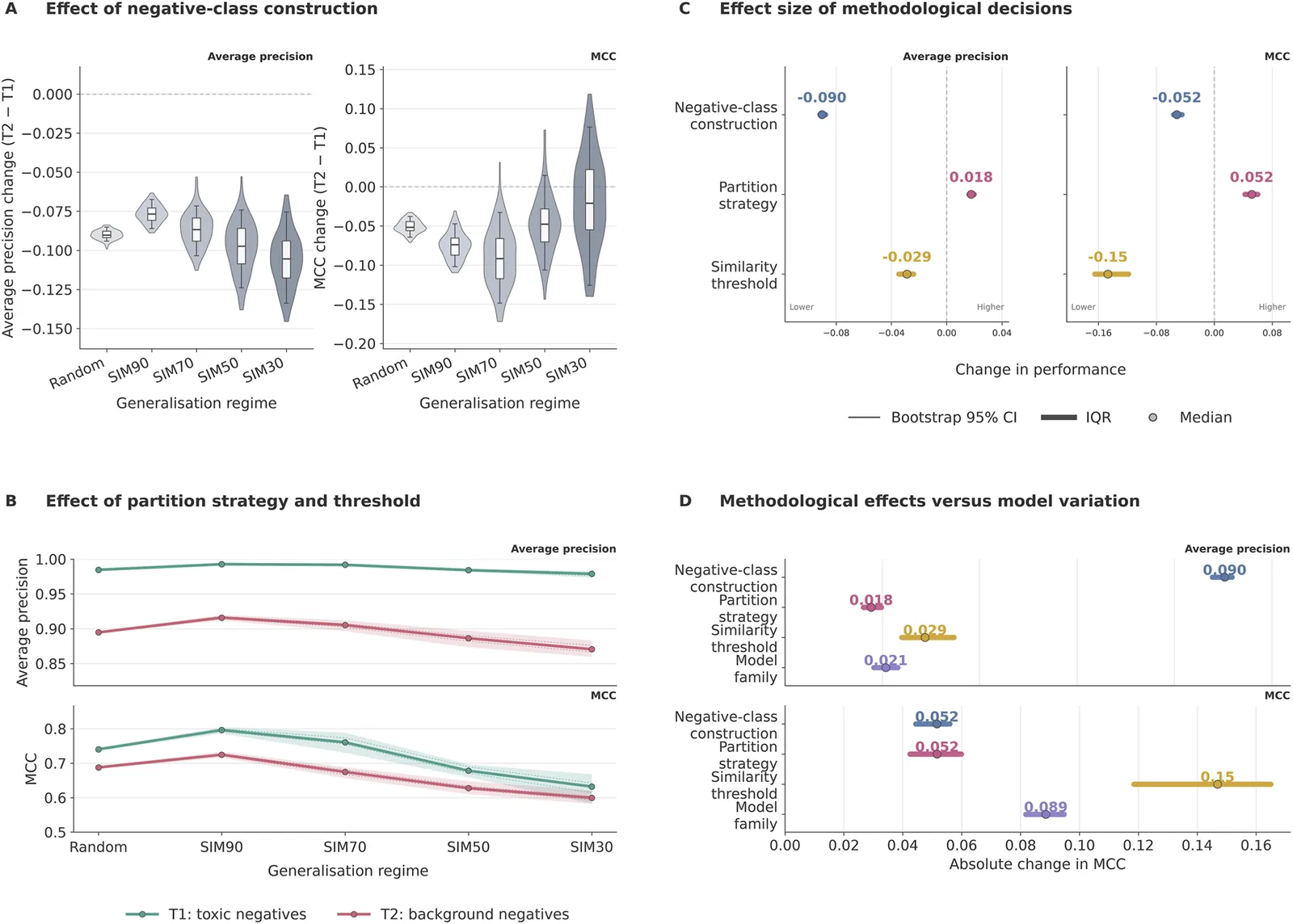
Workflow decisions reshape apparent performance in antimicrobial peptide classification. **(A)** Performance difference between the broad-background and toxic-background task across RANDOM and four similarity-aware regimes. Negative values indicate lower performance under the broader negative-class construction. **(B)** Model-aggregated test average precision and MCC across partitioning regimes for both tasks. **(C)** Predefined contrasts for negative-class construction, partition strategy, and the H30–H90 similarity-threshold comparison. Points show medians, thick intervals show interquartile ranges, and thin intervals show bootstrap 95% confidence intervals. **(D)** Absolute methodological contrasts compared with variation among the four model families. Results are descriptive and limited to the evaluated tasks, representation, classifiers, and partitioning regimes.

Figure 2A first isolates the observed difference between the two benchmark formulations within each partitioning regime. Replacing the toxic background with the broader candidate-negative background consistently reduced average precision, showing that two[the choice of negative-class definition and the resulting benchmark construction can substantially change the apparent difficulty of an AMP-classification benchmark.] Because the tasks also differed in prevalence, size, sequence composition, and physicochemical properties, this pattern should not be interpreted as the isolated causal effect of relabelling. Instead, it shows that studies described under the same AMP-classification label may encode meaningfully different prediction problems. Additional task-characterisation analyses are provided in SI Sections S3.1 and S3.4.

Figure 2B shows how test performance changed across the RANDOM and similarity-aware regimes for both task formulations. The relationship was not monotonic with increasingly stringent similarity control. In particular, H90 could outperform RANDOM, whereas more restrictive regimes reduced performance, demonstrating that similarity-aware evaluation is not inherently more difficult and cannot be interpreted as a single benchmark condition. Its effect depends on the clustering procedure, similarity threshold, resulting group structure, and exact partition assignments. Supporting analyses of similarity structure and partition feasibility are provided in SI Sections S3.2 and S3.5.

The predefined contrasts in Figure 2C quantify these patterns across the full set of repeated runs. Changing from the toxic to the broader negative-class construction produced a median contrast of (−0.090) in average precision and (−0.052) in MCC. Relative to RANDOM, H90 increased median average precision by 0.018 and MCC by 0.052, whereas moving from H90 to H30 reduced average precision by 0.029 and MCC by 0.150. The associated interquartile ranges and bootstrap 95% confidence intervals summarise the variation across the predefined analytical comparisons rather than a single train–test split.

Figure 2D places the magnitude of these methodological effects alongside variation among the four model families. Two[Within each task, regime, seed, and metric, model-family variation was calculated as the median absolute pairwise performance difference among the four classifier families and was subsequently aggregated across task–regime conditions at the seed level. For average precision, the median absolute change associated with negative-class construction was 0.090, compared with a model-family twovariation of 0.021. For MCC, the H30–H90 contrast reached 0.150, compared with a model-family twovariation of 0.089. twoBecause only four conventional classifier families were evaluated, this comparison is intended as an illustrative comparison of effect magnitudes rather than as a general estimate of algorithmic variability. These results do not establish a universal hierarchy between workflow and model effects, but show that benchmark construction and evaluation choices can produce differences comparable to or larger than those observed among the evaluated predictive algorithms. Additional model-family comparisons are reported in SI Sections S3.6–S3.8.

The case study therefore reinforces the central argument of this Perspective. Task semantics, negative-class construction, similarity control, and partition definitions are part of the benchmark itself. They determine what is being predicted, which form of generalisation is evaluated, and how performance should be interpreted. Without explicit records of these decisions, differences between studies may be incorrectly attributed to model quality or dataset difficulty. Scope and limitations are discussed in SI Section S3.9.

## 6 Outlook

The future reliability of protein machine learning will depend not only on model advances, but also on whether benchmark-defining decisions become explicit, verifiable, and comparable across studies. As protein language models, multimodal architectures, and generative systems expand the range of prediction and design tasks (Xiao et al., 2025; Valentini et al., 2023; Trinquier et al., 2021; Hsieh et al., 2025), differences in task semantics, similarity control, representation provenance, execution conditions, and evaluation regimes will become increasingly consequential. Our demonstrative case study shows that such choices can materially alter reported performance and should therefore be treated as part of the scientific result.

Adoption can proceed incrementally. A minimal implementation may include versioned task definitions, dataset and partition manifests, immutable configurations, checksums, environment records, and predictions linked to their evaluation outputs. More complex or decision-facing workflows can extend this foundation with formal schemas, workflow engines, persistent archives, automated validation, calibration, uncertainty analysis, and prospective experimental records. Implementations may differ across laboratories, but the biological meaning, provenance, compatibility, and verification status of workflow artefacts should remain recoverable.

Further work should test these practices across additional protein tasks, representations, data modalities, and generalisation settings, including temporal, family-level, species-aware, structure-aware, and prospective evaluations. Particular attention is needed for representation provenance and potential pretraining contamination, which cannot be addressed through supervised train–test separation alone. Community agreement on minimum records and verification checks could improve reproducibility and comparability without prescribing a specific software stack, helping protein machine learning move toward workflows whose results can be independently reconstructed, verified, and interpreted in their biological context (Wilkinson et al., 2016; Chen et al., 2025; Boeckhout et al., 2018).

## Data Availability

All code, configurations, partition assignments, sample-level predictions, analysis outputs, and instructions required to reproduce the demonstrative case study are openly available at https://github.com/kren-ai-lab/demonstrative_examples_architectural_good_practices. The starting peptide activity matrix was derived from Peptipedia v2.0 (Cabas-Mora et al., 2024), as described in the Supplementary Information. The workflow was verified in a clean environment and can be executed using the archived environment specification without undocumented local dependencies. The version associated with this article is release v0.1.0 at Git commit 86c4cc857259ef0ead56efcf87a27e963b2541b4 and is archived in Zenodo at https://zenodo.org/records/21810167. Source code is released under the MIT License, while documentation, results, and metadata are available under the Creative Commons Attribution 4.0 International License.
