# Supplementary material for: Architectural good practices for reproducible benchmarking in protein machine learning
Source: Front Bioinform. 2026 Sep 8;6:1925740. doi: 10.3389/fbinf.2026.1925740 (PMC13593872; doi:10.3389/fbinf.2026.1925740)
Supplement: Supplementary file 1 [file Supplementaryfile1.pdf]

Supplementary Information: Architectural Good Practices for Reproducible Benchmarking in Protein Machine Learning

Julián García-Vinuesa[1], Diego Fernández-Villegas[1], Michelle Soto-García[1], Xavier Cadet[2], Mehdi D. Davari[3], Frederic Cadet[4], Juan A. Asenjo[5], Roberto Uribe-Paredes[1,*], and David Medina-Ortiz[1,3,*]

[1]Departamento de Ingeniería en Computación, Universidad de Magallanes, Avenida Bulnes 01855, 6210427, Punta Arenas, Chile.
[2]Dartmouth College, Hanover, NH, 03755, USA
[3]Department of Bioorganic Chemistry, Leibniz Institute of Plant Biochemistry Weinberg 3, 06120 Halle, Germany.
[4] AI for Biologics, PEACCEL, Paris, France.
[5]Departamento de Ingeniería Química, Biotecnología y Materiales, Universidad de Chile, Beauchef 851, 8370456, Santiago, Chile.
* Corresponding Author: Roberto Uribe-Paredes (roberto.uribe@umag.cl)
* Corresponding Author: David Medina-Ortiz (david.medina@umag.cl)
[†]Work done whilst at Department of Computing, Imperial College London, UK

Supplementary contents

S1 Operational requirements and minimum compliance **2**
S1.1 Operational requirements for verifiable protein machine learning . . . 2
S1.2 Minimum compliance checklist . . . 5

S2 Supplementary Methods **8**
S2.1 Dataset provenance and raw data freeze . . . 9
S2.2 Sequence preprocessing and quality control . . . 9
S2.3 Task formulation and label semantics . . . 10
S2.4 Demonstrative analytical design and methodological scenarios . . . 11
S2.5 Dataset composition and physicochemical characterisation . . . 11
S2.6 Redundancy and sequence-similarity analysis . . . 12
S2.7 Partition construction and validation . . . 13
S2.8 Representation generation . . . 14
S2.9 Predictive models and model selection . . . 15
S2.10 Evaluation and uncertainty analysis . . . 16
S2.11 Computational environment and execution records . . . 19
S2.12 Reproducibility and verification checks . . . 20

S3 Supplementary Results **21**
S3.1 Task and dataset characterisation . . . 21
S3.2 Similarity structure and partition feasibility . . . 22
S3.3 Model selection and execution integrity . . . 22
S3.4 Sensitivity to negative-class construction . . . 23
S3.5 Sensitivity to partitioning strategy and similarity threshold . . . 23
S3.6 Methodological contrasts relative to model-family variation . . . 24
S3.7 Absolute predictive performance and across-seed variability . . . 24
S3.8 Validation–test agreement and metric concordance . . . 25
S3.9 Scope and limitations of the demonstrative analysis . . . 25

S1 Operational requirements and minimum compliance

S1.1 Operational requirements for verifiable protein machine learning

The architectural requirements introduced in this work are operationalised here as a set of records, provenance relationships, and verification checks for protein machine learning workflows. Their purpose is to preserve the biological and computational meaning of workflow objects as they move across task definition, dataset construction, representation generation, partitioning, model development, execution, and evaluation. The requirements are implementation-agnostic and may be supported through different metadata formats, repositories, workflow systems, configuration languages, software environments, or provenance mechanisms. [Wilkinson et al., 2016, Leipzig et al., 2021, Antunes and Hill, 2024, Herrera-Rocha et al., 2025, Bernett et al., 2024, Heil et al., 2021]

A workflow object is any identifiable input, intermediate output, configuration, or result required to interpret or reconstruct an analysis. Examples include source datasets, curated datasets, sequence groups, partition assignments, learned representations, predicted structures, multiple-sequence alignments, trained models, prediction tables, evaluation summaries, execution records, and decision-facing outputs. Verifiability requires that these objects remain linked to the assumptions and conditions under which they were produced. A file alone is therefore insufficient when its biological meaning, version, configuration, or relationship to other workflow objects cannot be established.

The framework distinguishes requirements according to three levels of applicability and implementation depth. Core requirements apply to all workflows intended to support reproducible benchmarking. Conditional requirements apply when the workflow includes a corresponding component, such as a pretrained representation model, a predicted structure, or predictions used to guide biological or experimental decisions. Advanced practices extend the minimum baseline through automated validation, immutable archival, cross-platform execution, prospective evaluation, formal provenance graphs, or linkage to experimental outcomes. These categories are intended to support incremental adoption and should not be interpreted as a certification scheme or a formally endorsed community standard.

Minimum compliance requires sufficient information to reconstruct the declared workflow, verify the identity and compatibility of its artefacts, and interpret the biological question being evaluated. It does not require every intermediate object to be stored indefinitely. Large or regenerable artefacts, such as embeddings or predicted structures, may instead be represented through stable identifiers, generating configurations, software versions, input checksums, and integrity records when reconstruction remains technically feasible. In contrast, non-regenerable objects, exact partition assignments, model-selection records, and sample-level predictions used to derive reported results should be preserved directly.

Under this framework, evidence supporting compliance may be distributed across the manuscript, Supplementary Information, dataset or model cards, machine-readable manifests, configuration files, versioned repositories, archived releases, or execution logs. Regardless of its location, this evidence should be accessible, internally consistent, and unambiguously linked to the workflow component and requirement it supports. Descriptive statements without corresponding artefacts, configurations, or execution records may aid interpretation, but do not by themselves establish verifiability.

Supplementary Table S1: Operational requirements for verifiable protein machine learning workflows.

Requirement	Minimum required record	Verification objective	Implementation and advanced extensions
Deterministic curation	Source resource and release; access date; retrieval query or accession list; inclusion and exclusion criteria; sequence-normalisation rules; permitted alphabet; duplicate handling; annotation and label semantics; treatment of missing or ambiguous evidence; negative-class construction; redundancy-control procedure	Establish that the curated dataset represents the declared biological task and can be reconstructed from its reported sources and rules. Confirm identifier validity, sequence validity, duplicate status, class composition, label consistency, and provenance completeness.	Versioned curation scripts, machine-readable manifests, dataset cards, FAIR metadata, and quality-control reports provide a practical baseline. Advanced extensions include ontology-supported labels, automated source comparison, continuous curation validation, and explicit tracking of changes between dataset releases.
Persistent artefacts	Stable or persistent identifier; artefact type; semantic version or release identifier; checksum; generating configuration; execution identifier; creation date; upstream dependencies; downstream relationships; storage or retrieval location	Establish the identity and integrity of each artefact and determine whether it is compatible with the workflow stage in which it is used. Confirm that datasets, representations, partitions, models, predictions, and results can be traced through a complete lineage.	Versioned repositories, archived releases, persistent identifiers, provenance records, and dataset or model cards support minimum compliance. Advanced extensions include immutable storage, retention policies, content-addressable storage, formal provenance graphs, and automated invalidation of dependent artefacts when upstream objects change.
Explicit interface contracts	Required identifiers and fields; data types; dimensions; sequence or representation format; allowed values; missing-value policy; identifier conventions; compatibility constraints; expected input and output behaviour; schema or contract version	Establish that an artefact satisfies the structural and semantic assumptions of the downstream component that consumes it. Detect missing fields, invalid values, identifier mismatches, dimension changes, and incompatible representation or model versions before execution.	JSON Schema, typed interfaces, tabular schemas, workflow specifications, and the Common Workflow Language may be used. Advanced extensions include automated contract testing, schema migration rules, compatibility matrices, and validation across independent implementations.

Continued on the next page.

*Supplementary Table **S1** continued from the previous page.*

**Requirement**	**Minimum required record**	**Verification objective**	**Implementation and advanced extensions**
Reproducible execution	Software and dependency versions; environment or container definition; operating system; hardware context; numerical precision; random seeds; runtime parameters; parallelisation settings; accelerator configuration; relevant nondeterministic operations; execution command or workflow entry point	Establish that the computational state of an analysis is sufficiently specified to rerun the workflow and associate each result with the conditions under which it was produced. Distinguish expected stochastic variation from changes caused by environment or runtime drift.	Environment manifests, dependency lockfiles, containers, workflow engines, versioned configuration files, and execution logs provide baseline support. Advanced extensions include continuous integration, multi-platform reproduction, deterministic test suites, automated environment comparison, and output-level regression testing.
Transparent evaluation	Exact partition assignments; grouping or similarity rules; split sizes and class prevalence; metric definitions; class-dependent baselines; preprocessing and model-selection procedures; aggregation rules; number and identity of repeated runs; test-isolation conditions; prediction table used for metric calculation	Establish which biological generalisation regime was evaluated and whether the reported metrics can be independently recomputed. Confirm that partition constraints are satisfied, the test set remained isolated, and comparisons use compatible task definitions and evaluation protocols.	Versioned partition files, benchmark cards, evaluation manifests, reusable metric implementations, and sample-level prediction tables provide minimum compliance. Advanced extensions include temporal, family-level, structure-aware, external, prospective, and multi-site evaluation.
Decision readiness (conditional extension)	Sample-level prediction; model, representation, partition, configuration, and execution identifiers; score or probability; calibration record; uncertainty estimate; applicability information; explanation artefact when relevant; link to the decision or experimental action supported	Establish that each decision-facing prediction can be traced to its generating workflow and that its reliability can be assessed in relation to the intended use. Confirm output completeness and evaluate calibration, uncertainty, applicability, or explanation quality using task-appropriate procedures.	Prediction records, model cards, calibration reports, applicability assessments, and provenance-aware result tables provide a practical baseline. Advanced extensions include local and global explanation artefacts, decision logs, candidate-selection records, prospective validation, and linkage with experimental outcomes.

Deterministic curation establishes the meaning of the prediction problem before modelling begins. In protein machine learning, this includes not only the source sequences and labels but also the evidence rules through which labels were assigned. A negative label based on explicit experimental evidence is not equivalent to a label based on absence of annotation, sampling from an unrelated database, or generation of an artificial decoy [González-Cebrián et al., 2024, Youngs et al., 2014]. Consequently, dataset provenance must preserve both the origin of each observation and the semantic rules used to construct the task.

Persistent artefacts extend this traceability beyond the curated dataset. A trained model is interpretable only in relation to the representation, partitions, hyperparameters, and execution conditions used to generate it. Similarly, an evaluation table is meaningful only when it remains linked to the exact predictions and partition assignments from which its metrics were calculated. Checksums, stable identifiers, and explicit lineage links

provide practical mechanisms for preserving these relationships [Hartley and Olsson, 2020, Bayarri et al., 2024, Gundersen et al., 2022, Khan et al., 2019].

Interface contracts address a different problem. Two artefacts may be individually valid but mutually incompatible. A model trained using one embedding dimension, pooling strategy, or identifier order cannot safely consume a representation generated under different assumptions. Contracts therefore define the structural and semantic conditions that must hold at workflow boundaries. These conditions can be verified before execution and can prevent silent errors that would otherwise appear only as degraded or irreproducible performance [Kanwal et al., 2017, Amstutz et al., 2016, Meyer, 2002].

Reproducible execution preserves the computational state needed to regenerate workflow outputs. Recording a random seed alone is insufficient when dependency versions, numerical precision, hardware, parallelisation, or nondeterministic operations can affect the result. Conversely, bitwise identity should not be treated as universally achievable across all platforms and accelerators. The required level of reproducibility should therefore be stated explicitly, distinguishing exact output reproduction from statistically consistent behaviour across equivalent executions [Leipzig et al., 2021, Kurtzer et al., 2017, Merkel et al., 2014].

Transparent evaluation connects reported performance to the task and generalisation regime being assessed. Exact partition assignments are required because labels such as “random split”, “homology-aware split”, or “family-level evaluation” do not uniquely specify a benchmark. The corresponding grouping rules, thresholds, prevalence, baselines, model-selection protocol, and test-isolation conditions must also be preserved. Sample-level prediction tables are particularly valuable because they allow metrics, uncertainty summaries, and subgroup analyses to be recomputed independently [Kucera et al., 2023, Ferrer Florensa et al., 2024, Bernett et al., 2024, Pineau et al., 2021, Leipzig et al., 2021].

Decision readiness extends these requirements when predictions are used beyond benchmark comparison. Explanation artefacts are not required for every reproducible benchmark, and their inclusion should not be treated as evidence of reliability by itself. However, when predictions guide candidate prioritisation or experimental design, the workflow should preserve enough information to determine which model produced each prediction, under which conditions, with what level of uncertainty, and within which applicability domain [Leipzig et al., 2021, Doshi-Velez and Kim, 2017, Hie et al., 2020, Biswas et al., 2021].

S1.2 Minimum compliance checklist

The checklist in Supplementary Table **S2** provides a concise instrument for assessing whether the records required to interpret and verify a protein machine learning workflow are available. It is intended to support reporting, auditing, and independent review, not to assign a numerical reproducibility score. Each applicable item should be supported by accessible evidence rather than by a descriptive claim alone.

For practical use, an item may be classified as *complete*, *partial*, *not available*, or *not applicable*. Complete indicates that the required information and supporting artefact are available. Partial indicates that the workflow is described but one or more essential records, configurations, or checks are missing. Not available indicates that the information cannot be verified from the publication package. Not applicable should be used only when the corresponding workflow component is absent, for example when no pretrained model, predicted structure, or decision-facing output is used.

Minimum compliance requires all applicable core items to be complete. Conditional items are evaluated only when their triggering component is present. Advanced practices are not required for baseline compliance but may be reported to demonstrate additional robustness, portability, long-term preservation, or decision readiness.

Supplementary Table S2: Minimum compliance checklist for verifiable protein machine learning workflows.

ID	Compliance item	Expected evidence	Applicability
C1	The biological prediction task, unit of observation, target variable, and meaning of each class or response value are explicitly defined.	Task specification, dataset card, or Methods section	Core

Continued on the next page.

*Supplementary Table **S2** continued from the previous page.*

ID	Compliance item	Expected evidence	Applicability
C2	The source resources, database releases or access dates, retrieval procedures, search queries, accession lists, inclusion criteria, and exclusion criteria are recorded.	Dataset manifest and provenance record	Core
C3	Sequence normalisation, permitted residues, length restrictions, treatment of isoforms, duplicate handling, label harmonisation, and treatment of missing or ambiguous evidence are documented.	Curation configuration and quality-control report	Core
C4	The origin, evidence basis, and biological justification of the negative or background class are documented for classification tasks.	Task specification, dataset card, or label-assignment rules	Conditional on classification
C5	Class counts, prevalence, sequence-length distributions, and relevant compositional or physicochemical characteristics are reported for each task formulation.	Dataset summary and characterisation tables	Core for supervised tasks
C6	Redundancy, homology, or similarity-control procedures are reported with the software, version, parameters, thresholds, coverage rules, and grouping logic used.	Similarity configuration and grouping report	Conditional on similarity control
C7	The biological interpretation of each partitioning regime is stated, including which relationships between development and evaluation data are preserved or excluded.	Partition specification or benchmark card	Core
C8	Exact training, validation, and test assignments are available as versioned records, together with split sizes, class counts, and prevalence.	Partition files and partition metadata	Core
C9	Checks confirm that duplicates, prohibited similarity groups, or other constrained relationships do not cross partitions.	Partition-validation report	Core when constraints are declared
C10	The test set remains isolated from preprocessing decisions, feature selection, hyperparameter optimisation, model selection, threshold selection, and other development decisions.	Evaluation protocol and test-isolation check	Core
C11	Computationally derived inputs, including embeddings, alignments, predicted structures, or derived features, are linked to the software, model, database, version, parameters, and preprocessing configuration used to generate them.	Representation or inferred-input metadata	Conditional on derived inputs
C12	For protein language model representations, the model family, checkpoint, selected layer, tokenisation, special-token handling, pooling strategy, truncation policy, numerical precision, and output dimensionality are recorded.	Representation configuration	Conditional on protein language models
C13	For homology searches or multiple-sequence alignments, the reference database, database release, software, scoring system, thresholds, filtering rules, and alignment parameters are recorded.	Search or alignment configuration	Conditional on homology or MSA inputs
C14	For predicted structures, the generating model or resource, version, input sequence, confidence information, preprocessing procedure, and structure-selection rules are recorded.	Structural-input metadata	Conditional on predicted structures

Continued on the next page.

*Supplementary Table **S2** continued from the previous page.*

ID	Compliance item	Expected evidence	Applicability
C15	Potential pretraining contamination is acknowledged when pretrained models are used, and available information on model version, training-data chronology, sequence overlap, or close-homologue exposure is reported.	Representation metadata and limitations statement	Conditional on pretrained models
C16	Each persistent artefact has an identifier, version, checksum or integrity record, generating configuration, and links to its upstream dependencies.	Artefact manifest or provenance record	Core
C17	The relationship between datasets, representations, partitions, models, predictions, evaluations, and reported figures or tables can be reconstructed.	Result manifest, provenance graph, or figure-to-output mapping	Core
C18	Interfaces between workflow stages define required identifiers, fields, data types, dimensions, allowed values, missing-value policies, and compatibility constraints.	Schema, contract, or interface specification	Core for modular workflows
C19	The computational environment records the operating system, software versions, dependencies, hardware context, numerical precision, random seeds, runtime parameters, and relevant sources of nondeterminism.	Environment files, container, and execution records	Core
C20	The public execution procedure identifies the workflow entry point, required inputs, expected outputs, and commands needed to reproduce the analysis or regenerate reported results.	README, workflow definition, or executable script	Core
C21	The model-development procedure documents preprocessing, scaling, feature selection, hyperparameter spaces, search strategy, optimisation criterion, validation design, and final model-selection rule.	Model configuration and selection report	Core for predictive modelling
C22	It is stated whether models were retuned independently for each task, representation, partitioning regime, or experimental condition.	Model-selection protocol	Core when conditions are compared
C23	Metric definitions, prevalence-dependent baselines, threshold-selection rules, aggregation procedures, and uncertainty summaries are explicitly reported.	Evaluation manifest or statistical-analysis description	Core
C24	The number and identity of repeated runs or random seeds are recorded, and paired comparisons are preserved when equivalent configurations are compared.	Execution manifest and result table	Core when repeated runs are used
C25	Reported metrics can be independently recomputed from stored sample-level predictions and labels.	Prediction table and metric implementation	Core
C26	Reported conclusions remain limited to the evaluated tasks, datasets, representations, models, and generalisation regimes.	Results interpretation and limitations statement	Core
C27	The publication package provides a stable release, commit identifier, persistent archive or DOI, license, dependency specification, and citation information.	Repository release and archival record	Core
C28	Installation and execution instructions have been verified from a clean environment without undocumented local paths, unavailable files, or manual steps.	Clean-environment execution report or continuous-integration record	Core

Continued on the next page.

*Supplementary Table **S2** continued from the previous page.*

ID	Compliance item	Expected evidence	Applicability
C29	Large artefacts that are not archived directly can be regenerated from preserved inputs, configurations, software versions, and integrity records.	Regeneration workflow and artefact manifest	Conditional on regenerable artefacts
C30	Sample-level predictions preserve their model, representation, partition, configuration, and execution provenance when they are used to support biological or experimental decisions.	Prediction records and decision-facing output tables	Conditional on decision-facing use
C31	Calibration, uncertainty, and applicability are evaluated when they are relevant to the intended downstream decision.	Calibration report, uncertainty analysis, or applicability assessment	Conditional on decision-facing use
C32	Explanation artefacts are included only when justified by the model, analytical design, and intended use, and remain linked to the predictions and model version they describe.	Explanation records and provenance links	Conditional on explainability
C33	When computational predictions guide experiments, candidate-selection decisions and prospective experimental outcomes are linked to the corresponding prediction records where feasible.	Decision log and prospective validation record	Advanced

The checklist should be interpreted together with the biological and computational scope of the workflow. A small sequence-classification benchmark and a large multimodal protein-design pipeline may satisfy the same architectural requirements through substantially different implementations. The former may rely on tabular manifests and versioned configuration files, whereas the latter may require object storage, workflow engines, formal schemas, and distributed execution records. Compliance therefore concerns the completeness and verifiability of the relationships, not the use of a particular technology.

Absence of an advanced mechanism does not imply that a workflow is irreproducible. For example, a workflow may be verifiable without a formal provenance graph when equivalent lineage is preserved through stable identifiers, manifests, and explicit references. Conversely, the use of containers, persistent identifiers, or workflow engines does not establish compliance when task semantics, partition assignments, or artefact relationships remain undocumented.

The checklist is applied to the demonstrative antimicrobial peptide classification workflow in the following Supplementary sections. Those sections provide the task definitions, dataset records, similarity analyses, partition manifests, representation configuration, model-selection protocol, execution environment, evaluation procedures, and verification outputs used to support the case study.

S2 Supplementary Methods

The demonstrative antimicrobial peptide classification study was designed to show how methodological decisions that are frequently left implicit or incompletely documented can materially change reported benchmark performance. These choices can produce apparently inflated or attenuated estimates, alter conclusions about model behaviour and generalisation, and prevent the independent reconstruction or meaningful comparison of results across studies. The case study therefore examined three decisions that are particularly relevant to protein and peptide machine learning: the construction of the negative or background class, the use of conventional random or sequence-similarity-aware partitions, and the sequence-identity threshold used to define indivisible groups. By keeping the sequence representation, model-selection rules, evaluation framework, classification thresholds, and estimator-level random seed controlled, the analysis quantified how changes in these benchmark-defining decisions affected performance distributions across repeated partitions and conventional model families.

The workflow was organised as a sequence of configuration-driven and independently verifiable stages comprising raw-data freezing, deterministic sequence validation, task construction, dataset characterisation,

sequence-similarity analysis, partition generation, representation extraction, model-variant selection, held-out evaluation, and descriptive analysis of predefined methodological contrasts. A single frozen representation was used throughout, and one task-specific variant per model family was selected using the RANDOM validation regime before being frozen across all partitioning conditions. Each stage generated its principal output together with machine-readable records of its inputs, configuration, checksums, software context, and verification results. The complete configuration files, intermediate artefacts, exact partition assignments, sample-level predictions, analytical outputs, and execution manifests are provided in the associated repository and archival package.

S2.1 Dataset provenance and raw data freeze

The starting dataset was a peptide bioactivity matrix derived from Peptipedia v2.0 [Cabas-Mora et al., 2024]. The matrix had already been organised at the sequence level, with one record per unique peptide sequence and individual bioactivities represented as binary variables in separate columns. No sequence-level aggregation or duplicate-resolution operation was therefore required as part of the present case study.

The dataset was incorporated as the immutable file `data/raw/peptide_activity_pivot.csv`. It contained 86,477 unique peptide sequences and 214 columns, comprising one sequence field and 213 bioactivity-related variables. The biological sources, annotation harmonisation procedures, and construction of the underlying resource are described in the Peptipedia publication and its associated documentation [Cabas-Mora et al., 2024].

Before sequence normalisation or task-specific selection, the input file was characterised through a raw-file manifest. The manifest recorded the filename, file size, SHA-256 checksum, text encoding, field separator, sequence-column name, complete column structure, number of rows, and number of unique non-empty sequence values. These records describe the file as received, before any transformation performed for the present workflow.

The frozen input had a size of 39,947,062 bytes and the SHA-256 checksum

`4d4edbf35fea319844ec276939daf77aeb9e79a9994f0b44364fc68e63ed15c4`

The input checksum was propagated through downstream manifests to associate sequence preprocessing, task construction, physicochemical characterisation, similarity analysis, partition generation, and representation extraction with the same immutable source file.

The raw file was never modified in place. Sequence normalisation and all subsequent transformations generated separate artefacts under dedicated processed-data paths. The frozen input, its manifest, and the resulting workflow artefacts are included in the archived package described in the Data Availability Statement.

S2.2 Sequence preprocessing and quality control

Sequence preprocessing was performed deterministically using a YAML configuration. The original sequence value was retained in a dedicated field, whitespace and Unicode formatting characters were removed, and sequences were converted to uppercase. Sequence validity was assessed using the canonical 20-letter amino-acid vocabulary

$$\{\mathrm{A}, \mathrm{C}, \mathrm{D}, \mathrm{E}, \mathrm{F}, \mathrm{G}, \mathrm{H}, \mathrm{I}, \mathrm{K}, \mathrm{L}, \mathrm{M}, \mathrm{N}, \mathrm{P}, \mathrm{Q}, \mathrm{R}, \mathrm{S}, \mathrm{T}, \mathrm{V}, \mathrm{W}, \mathrm{Y}\}.$$

The source matrix was already valid under these criteria. All 86,477 sequences were non-empty, contained only canonical amino acids, and ranged from 2 to 150 residues, with a median length of 23 residues. No sequence was excluded because of missing content, unsupported characters, or sequence length, and no additional length filter was applied.

A stable identifier was generated for each sequence as the hexadecimal SHA-256 digest of its normalised amino-acid string. This identifier was used to link task records, physicochemical properties, similarity groups, partition assignments, representations, predictions, and evaluation outputs. Sequence length was calculated directly from the normalised value.

Quality-control checks confirmed that the 86,477 normalised sequences remained unique. No exact duplicates or conflicting antimicrobial and toxicity label profiles were identified. Because the source matrix already

contained one record per unique sequence, no deduplication or label-conflict resolution procedure was required before task construction.

S2.3 Task formulation and label semantics

Two binary antimicrobial peptide classification tasks were constructed from the same processed sequence matrix. Both tasks used the same positive class but differed in their definition of the negative or background class. The deliberate experimental change was therefore the construction of the comparison class and the resulting benchmark formulation.

Bioactivity annotations were obtained from the binary columns `Antimicrobial` and `Toxic`. All 86,477 sequences contained valid binary values in the `Antimicrobial` field. No record was excluded because of missing, ambiguous, or unsupported antimicrobial annotations.

For both tasks, the positive class was defined as

$$P = \{s \mid \texttt{Antimicrobial}(s) = 1\}.$$

These observations were assigned the binary label $y = 1$, recorded as `strict_positive`, and operationally interpreted as sequences with antimicrobial evidence in the source matrix. The shared positive class contained 34,410 unique sequences.

The first task, `amp_vs_toxic_without_amp_evidence`, used a constrained background composed of toxic peptides without antimicrobial evidence. Its negative class was defined as

$$N_{\text{toxic}} = \{s \mid \texttt{Antimicrobial}(s) = 0 \land \texttt{Toxic}(s) = 1\}.$$

A sequence was admitted to this negative class only when it lacked antimicrobial evidence and contained positive toxicity evidence in the source matrix. The resulting task contained 34,410 positive and 10,211 negative sequences, giving 44,621 sequences in total and a positive prevalence of 0.7712. A further 41,856 sequences with `Antimicrobial`=0 were not included because they lacked positive toxicity evidence.

This formulation compared antimicrobial peptides with peptides presenting another reported biological property. It therefore provided a narrower biological background than the complete collection of sequences without antimicrobial evidence.

The second task, `amp_vs_without_amp_evidence`, used all sequences without antimicrobial evidence as its background. Its negative class was defined as

$$N_{\text{broad}} = \{s \mid \texttt{Antimicrobial}(s) = 0\}.$$

This background contained the 10,211 toxic negative sequences used in the first task and an additional 41,856 sequences without reported antimicrobial activity. The resulting task contained 34,410 positive and 52,067 negative sequences, giving 86,477 sequences in total and a positive prevalence of 0.3979.

The relationship between the two background classes was

$$N_{\text{toxic}} \subset N_{\text{broad}}.$$

The first formulation assessed discrimination against a restricted background of peptides with toxicity evidence. The second assessed discrimination against a larger and more heterogeneous background consisting of all sequences without antimicrobial evidence.

Negative observations were recorded as `candidate_negative_background`, not as experimentally confirmed non-antimicrobial peptides. A value of zero in the `Antimicrobial` column was interpreted operationally as absence of antimicrobial evidence in the source matrix. It was not interpreted as proof that the sequence lacks antimicrobial activity under every biological or experimental condition.

The two tasks therefore represent distinct operational prediction problems. Their differences include negative-class meaning, task size, class prevalence, sequence composition, and physicochemical structure. They should not be treated as interchangeable versions of a single benchmark.

Each task record retained the task identifier, SHA-256 sequence identifier, normalised sequence, binary label, class definition, positive- and negative-class definitions, sequence length, source antimicrobial value, toxicity status, and contributing toxicity field. Because the source matrix contained one record per sequence, the sequence identifier was also used as the source-row identifier. Records were sorted deterministically by task identifier and sequence identifier.

The resulting long-format task table contained 131,098 task-specific records derived from 86,477 unique sequences. A task-construction report preserved the definitions, class counts, prevalence, and inclusion and exclusion outcomes associated with each formulation.

S2.4 Demonstrative analytical design and methodological scenarios

The case study was organised around three methodological scenarios whose consequences were quantified under a controlled representation and modelling procedure.

The first scenario concerned benchmark definition. The task `amp_vs_toxic_without_amp_evidence`, denoted T_{toxic}, was compared with `amp_vs_without_amp_evidence`, denoted T_{broad}. This comparison was used to quantify how alternative negative-class construction changed the resulting benchmark.

The comparison was not interpreted as the isolated causal effect of changing a label. Constructing the broader background also changed the size, prevalence, biological composition, physicochemical properties, and difficulty of the prediction problem. The corresponding contrast was therefore termed the *benchmark-definition contrast*.

The second scenario concerned partitioning strategy. The `RANDOM` regime provided a conventional stratified reference without sequence-similarity group constraints. H90, H70, H50, and H30 provided group-aware regimes in which sequences assigned to the same MMseqs2 [Steinegger and Söding, 2017] group were required to remain in the same partition.

The third scenario concerned the operational sequence-identity threshold used to construct those groups. Minimum identity thresholds of 0.90, 0.70, 0.50, and 0.30 were evaluated. Lower thresholds generally produced larger and coarser groups, thereby changing the sequence relationships permitted between development and evaluation data.

Four conventional classifier families were included as supporting analytical strata: k-nearest neighbours, logistic regression, random forest, and linear support vector machine. The classifiers were not the principal scientific comparison. They were included to determine whether sensitivity to task and partition construction was restricted to one modelling assumption or appeared across different inductive biases.

The complete experimental design comprised

$$2 \text{ tasks} \times 5 \text{ partitioning regimes} \times 4 \text{ model families} \times 100 \text{ partition seeds.}$$

The sequence representation, model-selection rule, metric definitions, classification thresholds, and estimator-level random seed were controlled. Representative model variants were selected independently for each task and model family and were subsequently frozen across all five partitioning regimes.

The primary analyses quantified three predefined methodological contrasts:

1. the benchmark-definition contrast between T_{broad} and T_{toxic};
2. the partition-strategy contrast between each similarity-aware regime and `RANDOM`; and
3. the identity-threshold contrast between H30 and H90, interpreted together with the complete H90–H70–H50–H30 trajectory.

The magnitude of these contrasts was also compared descriptively with the variation observed among the four model families. No assumption was made in advance that methodological sensitivity would be larger or smaller than model-family variation.

S2.5 Dataset composition and physicochemical characterisation

Dataset composition was characterised independently for each task and class. Descriptors were calculated once for each of the 86,477 unique sequences and linked to task-specific records through the stable sequence

identifier. Calculations were performed using modlAMP version 4.3.2 [Müller et al., 2017] under the archived configuration `configs/dataset_characterization.yml`.

The physicochemical descriptors were used only to characterise the datasets. They were not included as predictive variables in the classification models.

The analysed properties included sequence length, net charge, sequence-level hydrophobicity, hydrophobic-residue ratio, charged-residue proportion, and relative amino-acid composition. Net charge was calculated at pH 7.0 without assuming C-terminal amidation.

The hydrophobicity descriptor was generated using the Eisenberg scale with a configured window of 1,000 and the `max` aggregation modality. Hydrophobic-residue ratio was calculated using the corresponding modlAMP global descriptor.

Charged residues were identified operationally from the `charge_phys` scale using an absolute scale-value threshold of 0.0. Under this configuration, C, D, E, H, K, and R contributed to the charged-residue proportion. Amino-acid composition was represented as the relative frequency of each canonical residue.

Sequence lengths stored in the processed task table were compared with lengths independently calculated by modlAMP. Any disagreement caused execution to stop. Descriptor batches were additionally checked to ensure that the amino-acid ordering and charged-residue definition remained constant throughout execution.

Class distribution was reported as the number and fraction of positive and negative examples within each task. Sequence length and each physicochemical or compositional descriptor were summarised by task and class using the sample size, minimum, maximum, mean, sample standard deviation, median, first quartile, third quartile, and interquartile range.

This characterisation was used to determine how negative-class construction changed dataset composition and to provide context for the benchmark-definition contrast. Differences in length, charge, hydrophobicity, or composition were not treated as confounders to be removed from the analysis. They were consequences of the operational task definition under examination.

The characterisation was descriptive. No hypothesis tests were used to claim universal physicochemical differences between antimicrobial and background peptides. The sequence-level descriptor table, class-distribution table, length summary, physicochemical summary, and characterisation manifest are provided in the repository.

S2.6 Redundancy and sequence-similarity analysis

Sequence-similarity groups were generated separately for each task using MMseqs2 [Steinegger and Söding, 2017] version 13.45111. Clustering was applied directly to amino-acid sequences and not to the learned representations or physicochemical descriptors.

Positive and negative examples were clustered jointly within each task. Similarity relationships crossing class labels were therefore retained and could constrain downstream partition construction.

Four operational identity configurations were evaluated:

$$\mathrm{H90} = 0.90, \qquad \mathrm{H70} = 0.70, \qquad \mathrm{H50} = 0.50, \qquad \mathrm{H30} = 0.30.$$

All MMseqs2 executions used a minimum alignment coverage of 0.90, coverage mode 0, cluster mode 1, alignment mode 3, an E-value threshold of 10^{-3}, sensitivity 7.5, a maximum of 1,000 accepted sequences per query, and 16 threads. The minimum sequence-identity threshold was the only clustering parameter changed across H90, H70, H50, and H30.

For each task and identity configuration, sequences were exported to a deterministic FASTA file sorted by sequence identifier. Effective commands, optional arguments, software information, return codes, elapsed execution times, and output checksums were retained in stage-specific logs and manifests.

MMseqs2 membership was validated against the complete set of sequences expected for each task. Every sequence was required to occur in exactly one group. Missing identifiers, unknown identifiers, or multiple assignments caused execution to stop.

Stable group identifiers were generated from the SHA-256 hash of the task identifier, homology-configuration identifier, and sorted sequence identifiers belonging to the group. A shortened hash was used in the human-readable identifier, while the complete digest was retained in the manifest.

The clustering outputs recorded group membership, representative sequence, group size, positive and negative counts, positive fraction, mixed-label status, and sequence-length range. Dataset-level summaries included the number of groups, number and fraction of singletons, largest group size, mean and median group size, number of mixed-label groups, and number of sequences contained in mixed-label groups.

No sequence was removed because of similarity to another sequence. Similarity groups were instead treated as indivisible units during group-aware partition construction. This retained the complete task dataset while preventing members of the same operational MMseqs2 group from appearing in more than one partition.

Before definitive partition generation, each task–identity configuration underwent a structural and split-feasibility audit. The audit was independent of predictive performance and used 20 seeds, numbered 0–19, with up to 50 candidate group assignments evaluated for each seed. Candidate assignments targeted 70% training, 15% validation, and 15% test data.

The audit evaluated group counts and sizes, singleton fraction, largest-group fraction, mixed-label groups, sequences in mixed-label groups, class availability, and the ability to construct partitions with acceptable size and prevalence.

A diagnostic assignment was considered feasible when its maximum relative partition-size deviation did not exceed 0.10, its maximum absolute prevalence deviation did not exceed 0.05, and each partition contained at least 30 sequences, 10 positive examples, and 10 negative examples.

A configuration was classified as `eligible` when its largest group represented no more than 20% of the corresponding task dataset and at least 80% of the diagnostic seeds produced feasible assignments. Configurations failing the group-size requirement were classified as `structurally_problematic`. Configurations with insufficiently stable assignment feasibility were classified as `split_feasibility_unstable`. Only eligible configurations were used for definitive partition generation.

The implemented similarity control establishes that no MMseqs2 group crosses partitions. It does not calculate an exhaustive pairwise alignment between every sequence assigned to different partitions. H90, H70, H50, and H30 therefore identify operational MMseqs2 grouping configurations. They do not represent empirically verified upper bounds on all cross-partition pairwise identities.

S2.7 Partition construction and validation

Each task was partitioned under one conventional stratified-random regime and four MMseqs2 group-aware regimes. All regimes targeted 70% training, 15% validation, and 15% test data.

Definitive partition generation used 100 seeds, numbered 0–99. The complete partitioning design therefore generated

$$2 \text{ tasks} \times 5 \text{ regimes} \times 100 \text{ seeds} = 1{,}000$$

partition sets.

For the `RANDOM` regime, positive and negative sequence identifiers were handled separately. Within each class, identifiers were sorted and shuffled using the partition seed. Integer partition sizes and class counts were calculated using the largest-remainder method. This produced exact total sizes where possible and approximately preserved the task-level prevalence.

For H90, H70, H50, and H30, complete MMseqs2 groups were assigned to partitions and were never divided. Assignment used a deterministic greedy optimisation procedure that considered deviations from the target total size, positive count, and negative count in each partition.

The three objective components received equal weights of 1.0. Assignments exceeding their target counts received an additional overflow penalty of 2.0. Groups were processed in descending order of size and class concentration. Seed-controlled shuffling before sorting provided deterministic variation among otherwise equivalent ordering cases.

Up to 50 candidate assignments were evaluated for each partition seed. The feasible candidate with the lowest objective value was retained. When an eligible candidate had already been identified during the

structural audit, that attempt was reused and evaluated under the stricter definitive validation criteria. If it did not satisfy those criteria, the full configured attempt range was searched.

Every definitive partition set was required to satisfy the following conditions:

- every expected sequence occurred exactly once;
- no unexpected sequence identifier was present;
- training, validation, and test partitions were present and non-empty;
- every partition contained positive and negative examples;
- no MMseqs2 group crossed partitions in a group-aware regime;
- the maximum relative deviation from the target partition size did not exceed 0.02;
- the maximum absolute deviation from the task-level prevalence did not exceed 0.01;
- every partition contained at least 30 sequences, 10 positive sequences, and 10 negative sequences; and
- the 100 assignments were unique within each task and regime.

Each assignment table retained the task identifier, sequence identifier, label, partition name, regime identifier, operational identity threshold, group identifier, partition seed, and split identifier.

An assignment checksum was calculated from the sequence-to-partition mapping after sorting by sequence identifier. The split identifier was generated by hashing the task identifier, regime identifier, partition seed, and assignment checksum.

Deterministic regeneration was evaluated immediately after construction. The selected assignment was reconstructed using the same task, configuration, seed, and assignment attempt. The regenerated sequence-to-partition checksum was required to match the original checksum.

Partition assignments were stored as compressed CSV files. A partition index recorded the task, regime, seed, split identifier, assignment path, checksum, and validation status. A separate validation table retained partition sizes, positive and negative counts, prevalence, size deviations, prevalence deviations, and group-integrity results.

The partition-generation manifest linked each assignment to the task dataset, homology configuration, audit records, YAML configuration, and validation outputs.

Partition generation was completed before predictive model evaluation. Partition assignments were not used to select the pretrained representation. The representation was extracted once without fitting any task labels or partition-specific parameters.

S2.8 Representation generation

A single frozen representation asset was generated for the union of the 86,477 unique sequences appearing across the two tasks. The same representation was used for every task, partitioning regime, partition seed, and model family. Variation in representation generation was therefore excluded from the methodological contrasts.

Representations were generated using Sylphy version 0.2.0 and the pretrained ESM2 model [Lin et al., 2023, Medina-Ortiz et al., 2026]

`facebook/esm2_t6_8M_UR50D`.

The requested Hugging Face revision `main` was resolved before extraction to the immutable commit

`c731040fcd8d73dceaa04b0a8e6329b345b0f5df`

The model contains six transformer layers and produces a 320-dimensional hidden representation for each token.

The final transformer layer was selected. Padding and special tokens were excluded from pooling. The remaining residue-level vectors were averaged to obtain one 320-dimensional representation per sequence. Embeddings were generated in 32-bit floating-point precision.

The maximum permitted raw sequence length was 150 residues. Extraction was configured to stop when a sequence exceeded this value, and silent truncation was not permitted. Because all sequences contained between 2 and 150 residues, no sequence was truncated or excluded during representation generation. Tokenised inputs included two special tokens and therefore had a maximum length of 152 positions.

The requested batch size was 256. Automatic batch-size reduction was permitted only following a CUDA out-of-memory exception. The extraction seed was 42. TensorFloat-32 operations and cuDNN benchmarking were disabled, and deterministic cuDNN behaviour was requested.

Before extraction, each sequence identifier was compared with the SHA-256 digest of its normalised sequence. Each identifier was required to map to one sequence, and each normalised sequence was required to map to one identifier.

The final representation matrix was required to contain 86,477 rows and 320 embedding dimensions. All values were required to be finite and stored as `float32`. The output was sorted by sequence identifier and written to a Zstandard-compressed Parquet file.

The exported file was read back immediately. Identifiers, sequence ordering, row count, column count, numerical precision, and embedding values were compared with the in-memory representation. Any disagreement caused execution to stop.

The representation manifest recorded the input path and checksum, canonical sequence-table checksum, model repository, requested and resolved model revisions, model and tokenizer classes, hidden dimension, number of transformer layers, selected hidden state, special-token policy, pooling method, numerical precision, maximum sequence length, requested and effective batch sizes, extraction seed, software environment, hardware context, execution duration, output dimensions, and output checksum.

The supervised partition controls do not exclude the possibility that identical sequences or close homologues were encountered during ESM2 pretraining. Direct sequence-level overlap with the model's pretraining corpus was not quantified. The immutable model revision and available provenance were preserved, but potential pretraining exposure remains a limitation separate from supervised partition leakage.

S2.9 Predictive models and model selection

Four conventional classifier families were evaluated using the frozen 320-dimensional ESM2 representations: k-nearest neighbours, logistic regression, random forest, and linear support vector machine. The implementation used scikit-learn version 1.8.0. [Pedregosa et al., 2011]

The classifiers were selected to provide local distance-based, linear probabilistic, nonlinear ensemble, and linear maximum-margin decision rules. They were not evaluated to establish a state-of-the-art antimicrobial peptide predictor. Their role was to determine whether the effects of benchmark construction were consistent across different conventional modelling assumptions.

Standard scaling was applied to k-nearest neighbours, logistic regression, and linear support vector machine models. For each run, the scaler was fitted on the training partition and then applied to validation and test data. Random forests received the unscaled representations.

The candidate grid was declared explicitly in the archived modelling configuration and contained 30 variants: six k-nearest-neighbour variants, six logistic-regression variants, 12 random-forest variants, and six linear support vector machine variants.

The k-nearest-neighbour models used brute-force Euclidean search. Distance-weighted variants used

$$k \in \{3, 5, 15, 31, 63\},$$

and one additional variant used uniform weighting with $k = 15$.

Logistic regression used an L_2 penalty, the `lbfgs` solver, an intercept, a maximum of 5,000 iterations, and tolerance 10^{-4}. Balanced variants used

$$C \in \{0.01, 0.1, 1, 10, 100\},$$

and one unweighted variant used $C = 1$.

Random forests used 200 trees, the Gini criterion, bootstrap sampling, and parallel execution. The declared variants evaluated maximum depths of 10, 20, 40, or no depth limit; minimum leaf sizes of 1, 5, or 10; and feature-selection settings of `sqrt`, `log2`, 0.25, or 0.50. Eleven variants used balanced-subsample class weighting. One unrestricted-depth variant with a minimum leaf size of 5 and `sqrt` feature selection omitted class weighting.

The linear support vector machine used an L_2 penalty, squared-hinge loss, automatic primal or dual selection, an intercept, a maximum of 10,000 iterations, and tolerance 10^{-4}. Balanced variants used

$$C \in \{0.01, 0.1, 1, 10, 100\},$$

and one unweighted variant used $C = 1$. The linear support vector machine was not probability calibrated and produced decision-function scores.

Model-variant selection was separated from final comparison of the partitioning regimes. Every candidate variant was fitted on the training subset and evaluated on the validation subset of the `RANDOM` regime for each of the 100 reference seeds. The test subset associated with a reference split was not loaded during its grid evaluation, and test metrics were not used in variant ranking.

For each task and model family, one representative variant was selected using the median validation AP across the 100 RANDOM seeds. Variants were ranked by decreasing median validation AP, decreasing mean validation AP, increasing validation AP interquartile range, and lexical variant identifier. Candidates producing convergence warnings were rejected.

The selection stage comprised

$$2 \text{ tasks} \times 30 \text{ variants} \times 100 \text{ seeds} = 6{,}000$$

training and validation evaluations.

The selected task-specific variant for each model family was then frozen and applied without additional hyperparameter optimisation to RANDOM, H90, H70, H50, and H30. No model was retuned independently for a similarity regime or partition seed.

For each final run, preprocessing and the selected estimator were fitted on the training subset only. Models were not refitted using the combined training and validation data. The same trained estimator generated validation and test scores.

Probability-producing models used a fixed classification threshold of 0.5. The uncalibrated linear support vector machine used a decision-function threshold of 0.0. Thresholds were not optimised by task, regime, seed, validation partition, or test partition.

The estimator-level random seed was fixed at 20260731 across all partition sets. This reduced estimator-internal variation and made partition construction the principal source of variation among repeated runs.

The final modelling stage comprised

$$2 \text{ tasks} \times 5 \text{ regimes} \times 4 \text{ model families} \times 100 \text{ seeds} = 4{,}000$$

trained model executions. Each execution generated one validation and one test performance record, giving 8,000 partition-level evaluation records.

Sample-level test predictions retained the task, regime, partition seed, split identifier, model family, selected variant, sequence identifier, group identifier, true label, continuous score, score type, fixed threshold, and predicted label. These records permit independent reconstruction of the reported test metrics.

S2.10 Evaluation and uncertainty analysis

Evaluation focused on the sensitivity of benchmark conclusions to methodological decisions. Absolute performance remained available, but the primary analytical quantities were predefined contrasts between benchmark formulations and partitioning regimes.

Performance was calculated separately for validation and held-out test partitions. Average precision (AP), implemented through `average_precision_score`, was used as the primary threshold-independent ranking metric. Matthews correlation coefficient (MCC) was used as the primary threshold-dependent metric.

AP and MCC were analysed together because they represent complementary properties. AP evaluates the ordering of positive examples under class imbalance. MCC evaluates agreement between observed and predicted classes at the declared fixed threshold.

ROC area under the curve, balanced accuracy, F1 score, precision, recall or sensitivity, specificity, accuracy, prediction-positive fraction, and confusion-matrix counts were retained as complementary outputs. Brier score was calculated only for models returning probability estimates and was not applied to the uncalibrated linear support vector machine.

For each test partition, the expected AP baseline under an uninformative ranking was defined as its positive prevalence,

$$\pi = \frac{N_{\text{positive}}}{N}.$$

AP above baseline was defined as

$$\text{AP}^{+} = \text{AP} - \pi.$$

Raw AP was used as the primary threshold-independent performance measure in the predefined methodological contrasts. Because the two task formulations had substantially different prevalence, the corresponding random-ranking baseline and AP^{+} were reported as complementary quantities to contextualise performance relative to the baseline implied by each task formulation. AP^{+} was therefore not substituted for raw AP in the primary comparisons.

Let

$$M^{(q)}_{t,r,f,s}$$

denote test performance for metric q, task t, regime r, model family f, and partition seed s, where the primary contrast metrics were

$$q \in \{\text{AP}, \text{MCC}\}.$$

Absolute generalisation trajectories were summarised at the seed level by taking the median across model families,

$$\widetilde{M}^{(q)}_{t,r,s} = \text{median}_{f \in \mathcal{F}}\, M^{(q)}_{t,r,f,s},$$

where $\mathcal{F}$ contained the four classifier families. Family-specific summaries were retained to determine whether the aggregate trajectory concealed model-dependent behaviour.

The benchmark-definition contrast was first calculated within each model family and seed as

$$D^{(q)}_{\text{task},r,f,s} = M^{(q)}_{T_{\text{broad}},r,f,s} - M^{(q)}_{T_{\text{toxic}},r,f,s}.$$

The primary seed-level benchmark-definition effect was then defined as

$$\widetilde{D}^{(q)}_{\text{task},r,s} = \text{median}_{f \in \mathcal{F}}\, D^{(q)}_{\text{task},r,f,s}.$$

A negative value indicated lower measured performance for the broader benchmark formulation. This contrast describes the difference between the resulting benchmark pipelines. It does not isolate a causal effect of changing only the negative label because task composition, prevalence, size, physicochemical structure, and task-specific selected model variants also differed.

For each similarity-aware regime $h \in \{\mathrm{H90}, \mathrm{H70}, \mathrm{H50}, \mathrm{H30}\}$, the partition-strategy contrast was calculated within task, model family, and seed as

$$D^{(q)}_{\mathrm{partition},t,h,f,s} = M^{(q)}_{t,h,f,s} - M^{(q)}_{t,\mathrm{RANDOM},f,s}.$$

The corresponding model-aggregated seed-level contrast was

$$\widetilde{D}^{(q)}_{\mathrm{partition},t,h,s} = \mathrm{median}_{f\in\mathcal{F}}\, D^{(q)}_{\mathrm{partition},t,h,f,s}.$$

Positive values indicated that the similarity-aware regime produced a higher estimate than the RANDOM reference under the aligned analysis index. Negative values indicated a lower estimate. These values quantify changes in benchmark outcome and do not imply that one partitioning regime is intrinsically superior or universally more difficult.

The identity-threshold contrast was calculated as

$$D^{(q)}_{\mathrm{threshold},t,f,s} = M^{(q)}_{t,\mathrm{H30},f,s} - M^{(q)}_{t,\mathrm{H90},f,s},$$

with the model-aggregated value

$$\widetilde{D}^{(q)}_{\mathrm{threshold},t,s} = \mathrm{median}_{f\in\mathcal{F}}\, D^{(q)}_{\mathrm{threshold},t,f,s}.$$

This contrast summarised the difference between the lowest and highest operational identity thresholds. It was interpreted together with the complete H90–H70–H50–H30 trajectory because performance and group structure were not assumed to change monotonically.

Contrasts were aligned by task, regime, model family, and numerical partition seed as applicable. Seed alignment preserves the repeated-execution index used by the workflow. It does not imply that the compared tasks or regimes contained identical test sequences or identical MMseqs2 group structures.

[Variation among model families was quantified using absolute pairwise performance differences among the four evaluated classifier families. For each task, regime, seed, and metric, the condition-level model-family variation was defined as]

$$[V^{(q)}_{\mathrm{model},t,r,s} = \mathrm{median}_{\substack{f_i,f_j\in\mathcal{F}\\ f_i<f_j}} \left| M^{(q)}_{t,r,f_i,s} - M^{(q)}_{t,r,f_j,s} \right|.]$$

[With four classifier families, this calculation comprised six pairwise absolute differences within each task–regime–seed condition. A seed-level model-family variation was then obtained by taking the median across the two tasks and five partitioning regimes,]

$$[S^{(q)}_{\mathrm{model},s} = \mathrm{median}_{t,r}\, V^{(q)}_{\mathrm{model},t,r,s}.]$$

[The absolute magnitudes of the benchmark-definition, partition-strategy, and identity-threshold contrasts were compared descriptively with the resulting seed-level model-family variation. This analysis was intended only to compare effect magnitudes within the evaluated case study. Because it included four conventional classifier families, it should not be interpreted as a general estimate of algorithmic variability or as evidence that methodological decisions universally dominate model choice.]

For each metric m, the validation–test gap was retained as a secondary diagnostic and defined as

$$G_m = m_{\mathrm{validation}} - m_{\mathrm{test}}.$$

Positive values indicate higher validation performance, negative values indicate higher test performance, and values close to zero indicate agreement. Validation–test gaps were used to describe across-seed variability and possible validation optimism. They were not the primary measure of methodological sensitivity.

The partition seed was treated as the repeated-analysis unit. Individual peptide sequences were not treated as independent replicates for uncertainty estimation, and the four model families were not interpreted as independent biological observations.

Seed-level distributions were summarised using the number of observations, mean, sample standard deviation, minimum, first quartile, median, third quartile, maximum, and interquartile range,

$$\mathrm{IQR} = Q_{0.75} - Q_{0.25}.$$

Percentile bootstrap 95% confidence intervals were calculated for seed-level medians using 10,000 resamples and base bootstrap seed 20260802. Bootstrap resampling was performed over the 100 partition-seed values. For methodological contrasts, the contrast was calculated before resampling so that the declared task, regime, model-family, and seed alignment was preserved.

No p-values or null-hypothesis significance tests were used. The analysis was descriptive and demonstrative. Bootstrap intervals quantify uncertainty across the implemented partition seeds. They do not quantify uncertainty across alternative datasets, annotation procedures, representation models, experimental assays, biological populations, or external validation settings.

Before analysis, the complete matrix of 4,000 final model executions and 8,000 validation and test records was required. Each task–regime–model combination was required to contain exactly 100 seeds, and every final execution was required to contribute one validation and one test record.

Duplicate records, incomplete validation–test pairs, missing seeds, modelling failures, changes in the selected model variant, changes in fixed thresholds, or differences in test-set composition among model families evaluated on the same partition caused analysis to stop.

The principal figure and methodological summary tables were generated only after these completeness, alignment, and consistency checks passed. Absolute model rankings and full family-specific performance summaries were retained as supplementary outputs and were not treated as the central scientific result.

S2.11 Computational environment and execution records

The workflow was implemented in Python 3.12.12 under a Linux execution environment. The final software stack included NumPy 2.4.4 [Harris et al., 2020], pandas 3.0.2 [pandas development team, 2026], SciPy 1.17.1 [Virtanen et al., 2020], scikit-learn 1.8.0 [Pedregosa et al., 2011], PyTorch 2.11.0 [Ansel et al., 2024], transformers 4.48.1 [Wolf et al., 2020], huggingface-hub 0.36.2 [Inc, 2025], pyarrow 23.0.1 [Developers, 2026], PyYAML 6.0.3 [Simonov, 2025], modlAMP 4.3.2 [Müller et al., 2017], Sylphy 0.2.0 [Medina-Ortiz et al., 2026], MMseqs2 13.45111 [Steinegger and Söding, 2017], Matplotlib 3.10.8 [Hunter, 2007], IPython 9.12.0. [Team, 2026]

ESM2 representation extraction was executed using an NVIDIA GeForce RTX 2070 Super with 8 GB of device memory and compute capability 7.5. The corresponding manifest recorded 12 available CPU cores, the CUDA runtime, cuDNN version, operating-system platform, device index, accelerator name, and total device memory.

Each executable workflow stage received an explicit YAML configuration. The configurations defined input paths, expected fields, sequence vocabulary, task semantics, descriptor settings, MMseqs2 parameters, feasibility requirements, partition proportions, representation settings, candidate model variants, selection rules, fixed thresholds, evaluation metrics, and output paths.

Existing outputs were not replaced unless an explicit overwrite option was provided. Stages supporting interrupted execution validated the relevant configuration and input checksums before reusing existing outputs.

MMseqs2 commands, optional arguments, return codes, and elapsed execution times were retained in stage-specific logs. Representation extraction recorded start and completion timestamps, requested and effective batch sizes, selected hidden state, tokenisation limits, software versions, device information, execution duration, and output checksum.

Model executions recorded the modelling-configuration checksum, representation checksum, partition checksum, split identifier, selected model variant, estimator parameters, preprocessing rule, fit and scoring durations, software versions, warnings, prediction output, and run-level manifest. Failed executions generated structured records containing the task, regime, seed, model, error type, error message, traceback, and failure-record path.

The final analytical stage recorded the analytical constants, bootstrap configuration, software versions, input and output checksums, Git state, and execution timestamps. The final public package will identify the environment specification `environment.yml`, repository release v0.1.0, Git commit 86c4cc857259ef0ead56efcf87a27e963b2541b4, and Zenodo DOI `https://zenodo.org/records/21810167`.

S2.12 Reproducibility and verification checks

Verification was integrated into the workflow at each stage.

At the data level, the immutable raw-file checksum was recorded before transformation. Sequence normalisation preserved the original value and generated a stable identifier from the normalised sequence. The workflow verified that all 86,477 sequences were non-empty, canonical, unique, and between 2 and 150 residues.

Task construction validated the binary antimicrobial and toxicity states and generated both background definitions deterministically. The task report recorded the 34,410 shared positive sequences, 10,211 toxic-background negatives, 52,067 broad-background negatives, resulting task sizes, prevalence, and task-specific exclusions.

At the characterisation level, one descriptor record was required for every unique sequence. Reported sequence lengths were compared with lengths independently calculated by modlAMP. Amino-acid ordering and the configured charged-residue definition were required to remain constant across descriptor batches.

At the similarity-analysis level, every expected task sequence was required to occur exactly once in each MMseqs2 membership table. Task identifiers, configuration identifiers, sequence labels, sequence lengths, and membership sets were validated against the task dataset. Mixed-label groups were retained and were not divided or removed.

Clustering eligibility was evaluated before predictive modelling and without using performance information. Group structure, largest-group fraction, class availability, partition-size deviation, prevalence deviation, and assignment feasibility were assessed before definitive partition generation.

At the partition level, every expected sequence was required to occur exactly once in training, validation, or test. Assignments were checked for missing or unexpected identifiers, duplicate assignments, absent classes, excessive size deviation, excessive prevalence deviation, and groups crossing partitions. Every assignment received a checksum and stable split identifier. Deterministic regeneration was required to reproduce the same sequence-to-partition mapping.

The similarity-leakage control concerns integrity of the operational MMseqs2 groups. Exhaustive pairwise cross-partition identity was not calculated. H90, H70, H50, and H30 must therefore be interpreted using the documented clustering parameters.

At the representation level, the ESM2 revision was resolved to an immutable commit before extraction. Sequence identifiers, alphabet, sequence-to-identifier correspondence, maximum length, row count, output dimension, numerical precision, missing values, infinite values, sorting, and identifier coverage were validated. Parquet serialisation was verified through immediate readback and comparison with the in-memory representation.

Before modelling, the representation checksum, representation manifest, partition-index checksum, partition-generation manifest, individual assignment checksum, and shared task-dataset checksum were verified.

Preprocessing was fitted on training data only. Candidate variants were evaluated using training and validation data from the RANDOM reference regime. No test metric entered the variant-ranking procedure. One representative variant was frozen for each task and model family before comparison of the similarity regimes. No regime received independent hyperparameter or threshold optimisation.

Sample-level test predictions preserved their task, regime, model family, selected variant, partition seed, split identifier, sequence identifier, group identifier, true label, continuous score, score type, threshold, and predicted label. Reported partition-level metrics can therefore be reconstructed from the stored predictions.

At the analytical level, the benchmark-definition, partition-strategy, and identity-threshold contrasts were specified independently of their observed directions. Contrasts were calculated within model family before aggregation across models, preserving the intended analytical alignment.

The final-analysis workflow required 4,000 model executions and 8,000 validation and test performance records. Duplicate rows, incomplete validation–test pairs, missing seeds, modelling failures, inconsistent se-

lected variants, inconsistent thresholds, or different test compositions among model families caused execution to stop.

Summary tables and figure inputs were generated from machine-readable outputs and linked to the corresponding analysis configuration. The analytical manifest retained checksums for the source performance tables, partition records, model-selection records, contrast tables, summary tables, and figure data.

The workflow does not claim bitwise identity across arbitrary combinations of hardware, accelerator drivers, and software versions. Representation extraction controlled its seed, numerical precision, TensorFloat-32 use, cuDNN benchmarking, and deterministic cuDNN setting, while the observed execution context was preserved in the manifest.

Potential ESM2 pretraining exposure was not quantified because the complete sequence-level pretraining corpus was not reconstructed. This limitation is separate from the supervised partition controls applied in the present workflow.

No explanation method was included because the case study did not prioritise experimental candidates or support an individual biological decision. Sample-level scores, labels, thresholds, Brier scores where applicable, model provenance, representation provenance, and partition provenance were nevertheless retained as reusable evaluation artefacts.

These controls allow each reported methodological contrast to be traced from the immutable source matrix through sequence validation, task construction, dataset characterisation, similarity grouping, exact partition assignment, frozen representation generation, task-specific model selection, sample-level prediction, and final seed-level analysis.

S3 Supplementary Results

The final demonstrative analysis comprised two antimicrobial peptide classification tasks, five partitioning regimes, four classifier families, and 100 predefined partition seeds. This design produced 1,000 validated partition sets and 4,000 final model executions, each evaluated on validation and held-out test data. The expected matrix of 8,000 performance records was complete. No model-training failures were observed, and the selected model variants, score types, classification thresholds, and test-set compositions remained consistent with the predefined analysis configuration.

The results are organised around the methodological decisions examined in the case study: negative-class construction, partitioning strategy, and the sequence-identity threshold used to define similarity groups. Model-family results are reported to establish whether the observed benchmark sensitivity was restricted to a particular classifier, not to identify a universally superior antimicrobial peptide prediction model.

S3.1 Task and dataset characterisation

The frozen source matrix contained 86,477 rows and 214 columns, with one record per unique peptide sequence. All 86,477 sequences passed the configured alphabet and non-empty sequence checks. No exact duplicates or conflicting antimicrobial and toxicity label profiles were detected after normalisation. Sequence lengths ranged from 2 to 150 residues, with a median length of 23 residues.

Both task formulations shared the same 34,410 antimicrobial-positive sequences but differed in the construction of their candidate-negative background. The task `amp_vs_toxic_without_amp_evidence`, denoted T_{toxic}, included 10,211 toxic peptides lacking antimicrobial evidence as negative examples. It therefore contained 44,621 sequences and had a positive-class prevalence of 0.771.

The task `amp_vs_without_amp_evidence`, denoted T_{broad}, included all 52,067 sequences lacking antimicrobial evidence as candidate negatives. It contained all 86,477 sequences and had a positive-class prevalence of 0.398. Its background comprised the 10,211 toxic negative sequences used in T_{toxic} together with 41,856 additional sequences without antimicrobial evidence.

The alternative background definitions produced different sequence-length distributions. Antimicrobial-positive sequences had a median length of 23 residues and an interquartile range of 14–42 residues. Toxic peptides lacking antimicrobial evidence were generally longer, with a median of 34 residues and an interquartile range of 20–64 residues. The broader candidate-negative class had a median length of 23 residues and an interquartile range of 13–47 residues.

The largest descriptive physicochemical difference was observed for net charge. Antimicrobial-positive sequences had a median net charge of 2.994 at pH 7, compared with −0.005 for the toxic-negative background and 0.861 for the broader background. Median hydrophobic ratios were 0.400, 0.378, and 0.385 for the positive, toxic-negative, and broad-background classes, respectively. The median proportions of residues operationally classified as charged were 0.323, 0.351, and 0.266. Median Eisenberg-scale hydrophobicity values were comparatively similar, at −0.012, −0.024, and 0.012, respectively.

These results confirm that changing the negative-class definition altered not only class prevalence and dataset size, but also sequence-length structure, electrostatic properties, hydrophobic composition, and amino-acid composition. The two task formulations therefore represented distinct operational prediction problems rather than interchangeable versions of a single AMP benchmark.

S3.2 Similarity structure and partition feasibility

Sequence-similarity grouping produced progressively fewer groups as the minimum MMseqs2 identity threshold was reduced. For T_{toxic}, the number of groups decreased from 29,275 under H90 to 22,895 under H70, 20,045 under H50, and 19,170 under H30. The corresponding values for T_{broad} were 65,459, 55,318, 50,617, and 49,383 groups.

Most groups were singletons under every configuration. Singleton fractions ranged from 0.770 to 0.830 in T_{toxic} and from 0.831 to 0.882 in T_{broad}. The largest group represented at most 1.38% of T_{toxic} and 0.72% of T_{broad}, remaining substantially below the predefined structural acceptability threshold of 20%.

Lower identity thresholds increased the number of sequences contained in mixed-label groups. In T_{toxic}, mixed-label groups contained 392 sequences under H90, 1,040 under H70, 1,625 under H50, and 2,050 under H30. These values corresponded to 0.9%, 2.3%, 3.6%, and 4.6% of the task dataset, respectively.

In T_{broad}, the corresponding mixed-label group counts were 1,601, 3,365, 5,120, and 5,790 sequences, representing 1.9%, 3.9%, 5.9%, and 6.7% of the dataset. Lower identity thresholds therefore produced coarser groups containing a larger fraction of relationships crossing the class boundary.

All eight task–similarity configurations were classified as eligible during the structural audit. Feasible training, validation, and test assignments were obtained for all 20 audit seeds at every identity threshold. Predictive performance was not used to determine configuration eligibility.

Definitive partition generation produced 100 unique assignments for each task and regime, giving 1,000 partition sets in total. All assignments passed the predefined sequence-integrity, group-integrity, size, prevalence, and class-availability checks. No sequence was duplicated, omitted, or assigned to more than one partition, and no MMseqs2 group crossed partitions in any similarity-aware regime.

Test-set size and prevalence remained stable across partitioning regimes within each task. For T_{toxic}, median test-set sizes ranged from 6,693 to 6,694 sequences, with median positive prevalence between 0.7711 and 0.7713. For T_{broad}, median test-set sizes ranged from 12,971 to 12,972 sequences, with median prevalence between 0.3979 and 0.3994.

Consequently, the performance differences observed among RANDOM, H90, H70, H50, and H30 were not explained by large changes in test-set size or overall class prevalence. They arose in the context of changes in group structure, sequence composition, and the relationships retained between development and evaluation partitions.

S3.3 Model selection and execution integrity

The reference-regime model-selection stage comprised 6,000 training and validation evaluations. All candidate evaluations completed successfully, and no convergence-related failure prevented representative-variant selection. Selection was performed separately for each task and model family using median validation average precision across the 100 RANDOM partition seeds.

For T_{toxic}, the selected variants were distance-weighted k-nearest neighbours with $k = 31$, balanced logistic regression with $C = 0.1$, a balanced random forest with maximum depth 40 and minimum leaf size 1, and a balanced linear support vector machine with $C = 0.01$. Their median validation AP values during selection were 0.989, 0.981, 0.989, and 0.980, respectively.

For T_{broad}, the selected variants were distance-weighted k-nearest neighbours with $k = 15$, unweighted logistic regression with $C = 1$, the same balanced random-forest configuration, and an unweighted linear

support vector machine with $C = 1$. Their median validation AP values were 0.936, 0.862, 0.929, and 0.861, respectively.

Each selected variant was frozen across RANDOM, H90, H70, H50, and H30. The same variant, estimator parameters, preprocessing rule, score type, and classification threshold were retained across all 100 seeds and five partitioning regimes within each task and model family. All 4,000 final model executions completed successfully.

The absence of regime-specific retuning was important for interpreting the subsequent contrasts. Differences between partitioning regimes could not result from selecting a different model variant or threshold for a particular similarity condition.

S3.4 Sensitivity to negative-class construction

Negative-class construction materially changed the resulting benchmark estimates. Across every partitioning regime, T_{broad} produced lower raw AP than T_{toxic}. In the predefined model-aggregated synthesis, the median benchmark-definition contrast,

$$\text{AP}_{T_{\text{broad}}} - \text{AP}_{T_{\text{toxic}}},$$

was -0.090. The corresponding median MCC contrast was -0.052.

These contrasts describe the difference between the two resulting benchmark formulations. They should not be interpreted as the isolated causal effect of changing a class label because the broader background also changed task size, class prevalence, sequence composition, physicochemical structure, and the model variants selected for each task. The result nevertheless shows that two studies nominally framed as AMP classification can report materially different performance because their negative classes operationalise different biological comparisons.

Raw AP was used as the primary threshold-independent metric because it is the conventional benchmark quantity reported in the main analysis. Its interpretation, however, depends on class prevalence. The random-ranking AP baseline was approximately 0.771 for T_{toxic} and 0.398 for T_{broad}.

AP above baseline,

$$\text{AP}^{+} = \text{AP} - \pi,$$

was therefore retained as a complementary measure. It was not substituted for raw AP in the primary contrasts. Instead, it was used to show how observed ranking performance related to the baseline implied by each task formulation.

For example, under RANDOM, k-nearest neighbours obtained median raw AP values of 0.989 in T_{toxic} and 0.936 in T_{broad}. Although raw AP was lower in the broader task, the corresponding AP-above-baseline values were approximately 0.218 and 0.538. The broader task therefore produced a lower absolute AP but a larger improvement over its substantially lower prevalence baseline.

Raw AP and AP^{+} consequently answer different questions. Raw AP describes the absolute precision–recall performance observed for the benchmark as constructed, whereas AP^{+} provides complementary context about improvement above the task-specific random-ranking baseline. Reporting the latter does not remove the biological and compositional differences between the two tasks.

S3.5 Sensitivity to partitioning strategy and similarity threshold

Partitioning strategy produced a second source of benchmark variation. The direction of this change was not monotonic with the use or apparent stringency of sequence-similarity control.

In the primary model-aggregated analysis, H90 increased median test AP by 0.018 relative to RANDOM. The corresponding MCC contrast was 0.052. H90 therefore produced higher, not lower, performance estimates than the conventional stratified-random reference under the evaluated workflow.

This pattern was observed across individual model families. Relative to RANDOM, median H90 AP differences ranged from 0.006 to 0.010 in T_{toxic} and from 0.011 to 0.031 in T_{broad}. Median MCC differences ranged

from 0.044 to 0.090 and from 0.035 to 0.050, respectively. H90 produced higher median AP and MCC for every model in both tasks.

H70 also produced higher AP for all four models in T_{toxic} and for k-nearest neighbours and both linear models in T_{broad}. Random-forest AP decreased under H70 in the broader task, while random-forest MCC decreased under H70 in both tasks. H50 produced mixed results, including small positive or near-zero AP differences for some k-nearest-neighbour and linear models but reductions for the random forest and for most MCC comparisons.

H30 produced the clearest reduction relative to RANDOM. In T_{toxic}, median paired AP differences ranged from -0.011 to -0.002, while MCC differences ranged from -0.202 to -0.044. In T_{broad}, median AP differences ranged from -0.040 to -0.009, and MCC differences ranged from -0.115 to -0.063.

The predefined identity-threshold contrast between H30 and H90 produced a median AP difference of -0.029 and a median MCC difference of -0.150 in the primary model-aggregated synthesis. The magnitude of the threshold contrast was therefore larger than the contrast between H90 and RANDOM, particularly for MCC.

These results do not establish a universal ordering in which every similarity-aware partition is more difficult than a random split or in which lower identity thresholds necessarily produce a gradual decrease in performance. Each MMseqs2 configuration changed the resulting groups, the distribution of mixed-label relationships, and the sequences assigned to development and evaluation partitions.

H90 and H70 could therefore produce evaluation sets that were more separable than particular RANDOM assignments, whereas the coarser group structures induced by H50 and H30 more consistently reduced performance. The term "similarity-aware partitioning" does not define a single benchmark condition, and its interpretation requires the exact identity threshold, coverage rule, grouping configuration, and partition assignments.

S3.6 Methodological contrasts relative to model-family variation

[The magnitudes of the predefined methodological contrasts were compared descriptively with variation among the four model families. Within each task, regime, seed, and metric, model-family variation was defined as the median of the six absolute pairwise performance differences among the four evaluated classifiers and was subsequently aggregated across task–regime conditions at the seed level.]

For raw AP, the median absolute contrast associated with negative-class construction was 0.090, compared with a median model-family spread of 0.021. The absolute H90–RANDOM partition contrast was 0.018, and the H30–H90 identity-threshold contrast was 0.029.

For MCC, the absolute negative-class construction and H90–RANDOM contrasts were both 0.052. The median model-family spread was 0.089, while the H30–H90 identity-threshold contrast reached 0.150.

Negative-class construction therefore exceeded the observed model-family spread for raw AP, while the identity-threshold contrast exceeded it for MCC. The remaining methodological contrasts were smaller than or comparable to the corresponding model-family variation.

[These comparisons are intended as an illustrative comparison of effect magnitudes within the evaluated case study. Because only four conventional classifier families were included, the model-family variation should not be interpreted as a general estimate of algorithmic variability. The results nevertheless show that decisions defining the task and generalisation regime can produce changes of the same order as, or larger than, differences observed among the evaluated classifiers.]

S3.7 Absolute predictive performance and across-seed variability

Absolute performance remained high for several task, regime, and model combinations, but differed substantially between the two benchmark formulations.

For T_{toxic}, median test AP under RANDOM ranged from 0.980 for the linear models to 0.989 for k-nearest neighbours and random forest. Median MCC ranged from 0.679 to 0.804. Under H90, median AP ranged from 0.991 to 0.996, and MCC ranged from 0.742 to 0.894. Under H30, median AP remained between 0.978 and 0.979, whereas MCC declined to values between 0.602 and 0.637.

For T_{broad}, median RANDOM AP ranged from 0.860 for the linear support vector machine to 0.936 for k-nearest neighbours. Median MCC ranged from 0.628 to 0.762. Under H90, median AP ranged from 0.890

to 0.961, and MCC ranged from 0.664 to 0.812. Under H30, median AP ranged from 0.845 to 0.921, while MCC ranged from 0.564 to 0.690.

Across-seed variability increased as the operational identity threshold was reduced. In T_{toxic}, AP interquartile ranges were 0.001–0.002 under RANDOM and increased to 0.008–0.011 under H30. MCC interquartile ranges increased from approximately 0.009–0.011 under RANDOM to 0.062–0.108 under H30.

In T_{broad}, AP interquartile ranges increased from 0.003–0.005 under RANDOM to 0.017–0.028 under H30. MCC interquartile ranges increased from 0.007–0.009 to 0.027–0.039.

Lower identity thresholds therefore affected not only median performance but also the sensitivity of the reported estimate to the particular partition assignment. This increased dispersion was especially pronounced for MCC in the toxic-background task.

S3.8 Validation–test agreement and metric concordance

Median validation–test gaps were generally close to zero, providing no consistent evidence of validation optimism across tasks, regimes, and model families. AP gaps were typically below 0.01 in absolute magnitude.

The principal change under lower identity thresholds was increased dispersion rather than a uniform directional gap. In T_{toxic}, MCC gap interquartile ranges reached 0.126–0.210 under H30, compared with 0.014–0.017 under RANDOM. In T_{broad}, MCC gap interquartile ranges increased from approximately 0.010–0.013 under RANDOM to 0.054–0.081 under H30.

Model rankings depended on both the task and evaluation metric. Random forest had the highest median AP and MCC under RANDOM for T_{toxic}, whereas k-nearest neighbours ranked first by AP under every similarity-aware regime. It also ranked first by MCC under H90, H70, and H50, while logistic regression achieved the highest median MCC under H30.

In T_{broad}, k-nearest neighbours ranked first by both AP and MCC under all five partitioning regimes. These rankings are reported as descriptive results and were not treated as the central scientific outcome of the case study.

AP and MCC were positively associated across seeds but were not interchangeable. Median Pearson correlations across task–regime–model combinations were 0.883 for T_{toxic} and 0.854 for T_{broad}.

The weakest associations occurred for several RANDOM configurations. For logistic regression in T_{toxic}, Pearson and Spearman correlations were 0.350 and 0.376, respectively. These disagreements reflect the distinction between continuous-score ranking performance and fixed-threshold classification behaviour and support the joint reporting of AP and MCC.

S3.9 Scope and limitations of the demonstrative analysis

The case study was designed to demonstrate the operational consequences of task definition, negative-class construction, sequence-similarity control, partition construction, model selection, and evaluation reporting. It was not designed to identify a universally superior antimicrobial peptide classifier or to establish a general quantitative relationship between sequence identity and predictive performance.

The two tasks shared the same positive sequences and were not independent biological benchmark datasets. Their candidate-negative classes were derived from source annotations. In particular, T_{broad} represented sequences without antimicrobial evidence and should not be interpreted as a collection of experimentally confirmed non-antimicrobial peptides. The toxic-negative class provided a more constrained biological background but was also compositionally distinct from the positive class.

The benchmark-definition contrast is consequently a compound contrast between two resulting task formulations. It includes changes in negative-class semantics, dataset size, prevalence, sequence composition, physicochemical structure, and task-specific selected model variants. It should not be interpreted as the causal effect of changing only one annotation field.

Only canonical sequences between 2 and 150 residues were evaluated. A single frozen representation, obtained from the final hidden layer of ESM2-T6 using residue-level mean pooling, was used across all experiments. Results may differ with alternative protein language models, checkpoints, layers, pooling strategies, physicochemical encodings, or structure-derived representations.

The model comparison was limited to four conventional classifier families and explicitly selected variants. A fixed estimator seed was used so that the reported across-run variability primarily reflected changes in partition assignments. The analysis does not quantify additional variation arising from stochastic estimator initialisation, alternative representation generation, different hyperparameter-search strategies, or changes in computational hardware.

H90, H70, H50, and H30 describe operational MMseqs2 grouping regimes using a minimum alignment coverage of 0.90. The validation procedure confirmed that no operational group crossed partitions. It did not perform an exhaustive pairwise calculation of maximum identity between every sequence assigned to different partitions. The regime labels should therefore not be interpreted as exact empirical upper bounds on all cross-partition pairwise identities.

Potential exposure of evaluation sequences or their close homologues during ESM2 pretraining was not quantified. The model repository and immutable commit were preserved, but the complete sequence-level pretraining corpus was not reconstructed. Potential pretraining contamination therefore remains distinct from the supervised partition controls applied in this study.

Bootstrap intervals quantify uncertainty in the median across the 100 implemented partition seeds. They do not represent uncertainty across alternative source datasets, annotation procedures, representation models, experimental measurements, or external biological populations. No temporal, external, family-held-out, prospective, or experimental validation was performed.

Finally, the observation that H90 and H70 sometimes exceeded RANDOM should not be interpreted as evidence that similarity-aware evaluation is intrinsically easier or that random partitioning is inherently more conservative. It demonstrates that named partitioning strategies alter sequence relationships and test composition in ways that cannot be inferred from their labels alone.

Benchmark claims therefore require exact task definitions, negative-class rules, grouping parameters, partition assignments, class-dependent AP baselines, repeated-run summaries, and an explicit statement of the generalisation regime evaluated. Raw AP remains the primary ranking metric in this analysis, while AP^+ provides complementary prevalence-dependent context and should not be interpreted as a replacement for the reported raw performance estimate.

References

Peter Amstutz, Michael R Crusoe, Nebojša Tijanić, Brad Chapman, John Chilton, Michael Heuer, Andrey Kartashov, Dan Leehr, Hervé Ménager, Maya Nedeljkovich, et al. Common workflow language, v1. 0. 2016.

Jason Ansel, Edward Yang, Horace He, Natalia Gimelshein, Animesh Jain, Michael Voznesensky, Bin Bao, Peter Bell, David Berard, Evgeni Burovski, et al. Pytorch 2: Faster machine learning through dynamic python bytecode transformation and graph compilation. In *Proceedings of the 29th ACM international conference on architectural support for programming languages and operating systems, volume 2*, pages 929–947, 2024.

Benjamin Antunes and David RC Hill. Reproducibility, replicability and repeatability: A survey of reproducible research with a focus on high performance computing. *Computer Science Review*, 53:100655, 2024.

Genís Bayarri, Pau Andrio, Josep Lluís Gelpí, Adam Hospital, and Modesto Orozco. Using interactive jupyter notebooks and bioconda for fair and reproducible biomolecular simulation workflows. *PLOS Computational Biology*, 20(6):e1012173, 2024.

Judith Bernett, David B Blumenthal, Dominik G Grimm, Florian Haselbeck, Roman Joeres, Olga V Kalinina, and Markus List. Guiding questions to avoid data leakage in biological machine learning applications. *Nature Methods*, 21(8):1444–1453, 2024.

Surojit Biswas, Grigory Khimulya, Ethan C Alley, Kevin M Esvelt, and George M Church. Low-n protein engineering with data-efficient deep learning. *Nature methods*, 18(4):389–396, 2021.

Gabriel Cabas-Mora, Anamaría Daza, Nicole Soto-García, Valentina Garrido, Diego Alvarez, Marcelo Navarrete, Lindybeth Sarmiento-Varón, Julieta H Sepúlveda Yañez, Mehdi D Davari, Frederic Cadet, et al. Peptipedia v2. 0: a peptide sequence database and user-friendly web platform. a major update. *Database*, 2024:baae113, 2024.

Apache Arrow Developers. Python library for apache arrow. `https://pypi.org/project/pyarrow/`, 2026. Python package, version 23.0.1.

Finale Doshi-Velez and Been Kim. Towards a rigorous science of interpretable machine learning. *arXiv preprint arXiv:1702.08608*, 2017.

Alfred Ferrer Florensa, Jose Juan Almagro Armenteros, Henrik Nielsen, Frank Møller Aarestrup, and Philip Thomas Lanken Conradsen Clausen. Spanseq: similarity-based sequence data splitting method for improved development and assessment of deep learning projects. *NAR genomics and bioinformatics*, 6(3): lqae106, 2024.

Alba González-Cebrián, Michael Bradford, Adriana E Chis, and Horacio González-Vélez. Standardised versioning of datasets: a fair–compliant proposal. *Scientific Data*, 11(1):358, 2024.

Odd Erik Gundersen, Saeid Shamsaliei, and Richard Juul Isdahl. Do machine learning platforms provide out-of-the-box reproducibility? *Future Generation Computer Systems*, 126:34–47, 2022.

Charles R Harris, K Jarrod Millman, Stéfan J Van Der Walt, Ralf Gommers, Pauli Virtanen, David Cournapeau, Eric Wieser, Julian Taylor, Sebastian Berg, Nathaniel J Smith, et al. Array programming with numpy. *nature*, 585(7825):357–362, 2020.

Matthew Hartley and Tjelvar SG Olsson. dtoolai: Reproducibility for deep learning. *Patterns*, 1(5), 2020.

Benjamin J Heil, Michael M Hoffman, Florian Markowetz, Su-In Lee, Casey S Greene, and Stephanie C Hicks. Reproducibility standards for machine learning in the life sciences. *Nature methods*, 18(10):1132–1135, 2021.

Fabio Herrera-Rocha, David Medina-Ortiz, Fabian Mauz, Juergen Pleiss, and Mehdi D Davari. Best practices for machine learning-assisted protein engineering. *Journal of Chemical Information and Modeling*, 2025.

Brian Hie, Bryan D Bryson, and Bonnie Berger. Leveraging uncertainty in machine learning accelerates biological discovery and design. *Cell systems*, 11(5):461–477, 2020.

J. D. Hunter. Matplotlib: A 2d graphics environment. *Computing in Science & Engineering*, 9(3):90–95, 2007. doi: 10.1109/MCSE.2007.55.

Hugging Face Inc. Hugging face hub. `https://pypi.org/project/huggingface-hub/`, 2025. Python package, version 0.36.2.

Sehrish Kanwal, Farah Zaib Khan, Andrew Lonie, and Richard O Sinnott. Investigating reproducibility and tracking provenance–a genomic workflow case study. *BMC bioinformatics*, 18(1):337, 2017.

Farah Zaib Khan, Stian Soiland-Reyes, Richard O Sinnott, Andrew Lonie, Carole Goble, and Michael R Crusoe. Sharing interoperable workflow provenance: A review of best practices and their practical application in cwlprov. *GigaScience*, 8(11):giz095, 2019.

Tim Kucera, Carlos Oliver, Dexiong Chen, and Karsten Borgwardt. Proteinshake: Building datasets and benchmarks for deep learning on protein structures. *Advances in Neural Information Processing Systems*, 36:58277–58289, 2023.

Gregory M Kurtzer, Vanessa Sochat, and Michael W Bauer. Singularity: Scientific containers for mobility of compute. *PloS one*, 12(5):e0177459, 2017.

Jeremy Leipzig, Daniel Nüst, Charles Tapley Hoyt, Karthik Ram, and Jane Greenberg. The role of metadata in reproducible computational research. *Patterns*, 2(9), 2021.

Zeming Lin, Halil Akin, Roshan Rao, Brian Hie, Zhongkai Zhu, Wenting Lu, Nikita Smetanin, Robert Verkuil, Ori Kabeli, Yaniv Shmueli, et al. Evolutionary-scale prediction of atomic-level protein structure with a language model. *Science*, 379(6637):1123–1130, 2023.

David Medina-Ortiz, Diego Álvarez, and Julián García-Vinuesa. Sylphy: Protein sequence representation: Encoders, embeddings, and reductions. `https://pypi.org/project/sylphy/`, 2026. Python package, version 0.2.0.

Dirk Merkel et al. Docker: lightweight linux containers for consistent development and deployment. *Linux j*, 239(2):2, 2014.

Bertrand Meyer. Applying'design by contract'. *Computer*, 25(10):40–51, 2002.

Alex T Müller, Gisela Gabernet, Jan A Hiss, and Gisbert Schneider. modlamp: Python for antimicrobial peptides. *Bioinformatics*, 33(17):2753–2755, 2017.

The pandas development team. pandas-dev/pandas: Pandas (version v3.0.2) [computer software]. *Zenodo*, 2026.

F. Pedregosa, G. Varoquaux, A. Gramfort, V. Michel, B. Thirion, O. Grisel, M. Blondel, P. Prettenhofer, R. Weiss, V. Dubourg, J. Vanderplas, A. Passos, D. Cournapeau, M. Brucher, M. Perrot, and E. Duchesnay. Scikit-learn: Machine learning in Python. *Journal of Machine Learning Research*, 12:2825–2830, 2011.

Joelle Pineau, Philippe Vincent-Lamarre, Koustuv Sinha, Vincent Larivière, Alina Beygelzimer, Florence d'Alché Buc, Emily Fox, and Hugo Larochelle. Improving reproducibility in machine learning research (a report from the neurips 2019 reproducibility program). *Journal of machine learning research*, 22(164): 1–20, 2021.

Kirill Simonov. PyYAML: A full-featured yaml processing framework for python. `https://pypi.org/project/PyYAML/`, 2025. Python package, version 6.0.3.

Martin Steinegger and Johannes Söding. Mmseqs2 enables sensitive protein sequence searching for the analysis of massive data sets. *Nature biotechnology*, 35(11):1026–1028, 2017.

The IPython Development Team. Ipython: Productive interactive computing. `https://pypi.org/project/ipython/`, 2026. Python package, version 9.12.0.

Pauli Virtanen, Ralf Gommers, Travis E Oliphant, Matt Haberland, Tyler Reddy, David Cournapeau, Evgeni Burovski, Pearu Peterson, Warren Weckesser, Jonathan Bright, et al. Scipy 1.0: fundamental algorithms for scientific computing in python. *Nature methods*, 17(3):261–272, 2020.

Mark D Wilkinson, Michel Dumontier, IJsbrand Jan Aalbersberg, Gabrielle Appleton, Myles Axton, Arie Baak, Niklas Blomberg, Jan-Willem Boiten, Luiz Bonino da Silva Santos, Philip E Bourne, et al. The fair guiding principles for scientific data management and stewardship. *Scientific data*, 3(1):1–9, 2016.

Thomas Wolf, Lysandre Debut, Victor Sanh, Julien Chaumond, Clement Delangue, Anthony Moi, Pierric Cistac, Tim Rault, Rémi Louf, Morgan Funtowicz, Joe Davison, Sam Shleifer, Patrick von Platen, Clara Ma, Yacine Jernite, Julien Plu, Canwen Xu, Teven Le Scao, Sylvain Gugger, Mariama Drame, Quentin Lhoest, and Alexander M. Rush. Transformers: State-of-the-art natural language processing. In *Proceedings of the 2020 Conference on Empirical Methods in Natural Language Processing: System Demonstrations*, pages 38–45. Association for Computational Linguistics, 2020.

Noah Youngs, Duncan Penfold-Brown, Richard Bonneau, and Dennis Shasha. Negative example selection for protein function prediction: the nogo database. *PLoS computational biology*, 10(6):e1003644, 2014.
